# Comparative EEG study of neurodynamics upon olfactory stimulation in COVID-19 patients

**DOI:** 10.3389/fnhum.2025.1571477

**Published:** 2025-06-18

**Authors:** Mariia Chernykh, Ihor Zyma, Bohdan Vodianyk, Yaroslav Subin, Ivan Seleznov, Anton Popov, Ken Kiyono

**Affiliations:** ^1^Department of Physiology and Anatomy, Educational and Scientific Center “Institute of Biology and Medicine”, Taras Shevchenko National University of Kyiv, Kyiv, Ukraine; ^2^School of Engineering, University of Málaga, Málaga, Spain; ^3^Instituto de Ingeniería Oceánica (IIO), Málaga, Spain; ^4^Department of Electronic Engineering, Igor Sikorsky Kyiv Polytechnic Institute, Kyiv, Ukraine; ^5^Division of Bioengineering, Graduate School of Engineering Science, Osaka University, Osaka, Japan; ^6^Faculty of Applied Sciences, Ukrainian Catholic University, Lviv, Ukraine

**Keywords:** electroencephalography, olfaction, COVID-19, power spectrum density, detrended moving average, cognitive impairment

## Abstract

Cognitive disturbances following COVID-19 have been widely reported, yet the neural dynamics underpinning such phenomena remain incompletely understood. This exploratory study examined cortical neurodynamics using electroencephalographic (EEG) analysis in three groups: individuals with severe COVID-19 (Group S), individuals recovered from moderate COVID-19 (Group M), and healthy controls (Group H). EEG recordings were obtained during the resting state and exposure to three odorants—ammonia (trigeminal), isoamyl acetate (olfactory), and mountain pine (mixed)—to assess reactivity under different sensory conditions. Power Spectral Density (PSD) and detrended moving average (DMA) analyses were applied to quantify both spectral power and long-range temporal correlations, respectively. Group S showed consistently elevated *β*-band PSD and *α*-scaling exponent values across all conditions, indicative of globally rigid and hyperexcitable dynamics. Group M exhibited partially recovered oscillatory patterns, including α3 enhancements, without statistically significant stimulus-driven modulation. Group H maintained physiologically typical EEG responses with limited olfactory reactivity. While these results suggest differential patterns of neurodynamic adaptation and rigidity among groups, interpretations regarding cognitive status remain tentative due to the absence of behavioral or neuropsychological testing. The findings underscore the utility of DMA as a complementary EEG analysis tool and provide a basis for hypothesis-driven research on post-COVID cortical reorganization. Future studies incorporating direct cognitive measures are essential to validate EEG-based biomarkers of brain function.

## Introduction

Recent studies have reported cognitive impairment in individuals recovering from COVID-19 (Chehrehnegar et al., 2019; Leonel Tavares-Júnior et al., 2022; Ceban et al., 2022), pointing to a growing interest in identifying effective diagnostic and treatment strategies. Large-scale cognitive studies have documented significant decrements in executive function, attention, and memory that persist months after infection, with severity correlating with the initial intensity of respiratory symptoms (Hampshire et al., 2021). Various central nervous system manifestations have been documented in relation to COVID-19 (Herman et al., 2020; Nath, 2020; Hwang et al., 2021), although the mechanisms underlying these effects remain under investigation. Cognitive decline in neurodegenerative conditions typically progresses slowly, often becoming clinically apparent only in the later stages of the disease.

Available clinical reports have suggested that COVID-19 may influence various physiological systems, including the nervous and autonomic systems (Méndez et al., 2021; Poletti et al., 2021). Some studies have described potential impacts on myelin integrity, homeostasis, and systemic organ function. While such observations raise important questions about the broader effects of the virus, further research is needed to establish the extent and nature of these outcomes.

Cognitive impairment has been reported as one of the most persistent post-COVID symptoms in a subset of patients, sometimes lasting for months after recovery. Neuroimaging studies have documented both structural and functional alterations in brain architecture that correlate with cognitive deficits in these patients, suggesting potential neurobiological mechanisms for post-COVID cognitive sequelae (Díez-Cirarda et al., 2022; Paolini et al., 2022; Thye et al., 2022). Nevertheless, systematic studies of neuroelectrical dynamics in this context remain relatively scarce, possibly due to methodological constraints during the pandemic period.

EEG findings in individuals with COVID-19 or long COVID have been described as non-specific, often including diffuse slowing, low-voltage activity, and occasional epileptiform abnormalities (Antony and Haneef, 2020). Some anomalies have been noted in frontal brain regions, which might correspond with proposed routes of viral invasion through olfactory and oral mucosa toward orbitofrontal structures. Other areas potentially affected include the limbic system, thalamus, hypothalamus, and temporo-parieto-occipital cortices. The identified pathway starts from the mucous membrane of the nasal cavity and oral cavity, leading to the olfactory bulbs and then into the orbitofrontal and frontal areas of the cortex. The piriform cortex, the limbic system as a whole, the thalamus, the hypothalamus, as well as separate areas of the neocortex, including the temporo-parieto-occipital regions (TPO) of both hemispheres, the brain stem, etc., may also be involved (forming individual affected loci). Clinicians associate such disorders with the severity of the disease. Notably, only nearly half of the patients reported further condition improvement (Lamontagne et al., 2021). The clinical significance of such findings continues to be examined, and interpretations should be cautiously approached, given the heterogeneity of patient presentations.

Olfactory stimulation was selected as a probe for cognitive assessment in this study due to its established association with neurocognitive processing. Prior studies have demonstrated a link between olfactory identification and cognitive status (Devanand et al., 2008), and olfactory decline has been observed in the early stages of neurodegenerative diseases such as Alzheimer’s (Uchida et al., 2020). To examine this, three odorants were employed: 10% ammonia solution (trigeminal stimulant), isoamyl acetate (olfactory), and *Pinus mugo* essential oil (mixed stimulus) (Petrova et al., 2008; Chaput et al., 2012; Fung et al., 2021).

In this study, EEG recordings were obtained from three groups of participants: individuals undergoing a severe course of COVID-19 at the time of recording (Group S), individuals who had experienced moderate illness and recovered (Group M), and healthy controls (Group H). EEG signals were acquired during the resting state and olfactory stimulation. None of the participants reported a history of seizure activity or showed patterns suggestive of epilepsy during the recordings.

Two analytical methods were employed: (a) the conventional power spectrum density (PSD) analysis approach, which represents the relative contribution of each frequency to the total EEG signal power from each electrode at each time point (Gu et al., 2022); (b) the detrended moving average algorithm (DMA) which is used to estimate the scaling exponent for long-range correlated time series (Carbone and Kiyono, 2016), including such time series as EEG (Seleznov et al., 2019). The DMA is widely used to estimate long- and short-term correlations of random time series, both one-dimensional and high-dimensional, in time and space domains (Gu and Zhou, 2010).

## Materials and methods

In total, 51 volunteer subjects (36 female and 15 male subjects; aged 45–70) participated in the study. They were divided into three groups based on the severity of the COVID-19 symptoms and the course of the disease: severe illness (Group S, Sievierodonetsk City Multidisciplinary Hospital, acute stage of disease; *n* = 20 (13 female and 7 male subjects), moderate illness (Group M, Taras Shevchenko National University of Kyiv, no hospital admission, 2–3 months after recovery (negative PCR-based test result); *n* = 21 (18 female and 3 male subjects), and healthy control group (Group H, negative PCR results, no history of COVID-19 symptoms, Taras Shevchenko National University of Kyiv *n* = 10 (5 female and 5 male volunteers). Subjects from all three groups reported no history of neurological or psychiatric disorders. Groups S and M patients reported olfactory dysfunction related to the coronavirus disease.

It is important to acknowledge two limitations in the sample composition that may impact the interpretation of our findings. First, the control group (Group H, *n* = 10) was smaller than both clinical groups (Group S, *n* = 20; Group M, *n* = 21). While this group size was sufficient for initial comparisons, the limited number of participants may reduce statistical power for detecting subtle between-group differences, particularly in the presence of inter-individual variability. Second, there was a notable gender imbalance, particularly in Group M (18 females, 3 males), which may introduce potential gender-related bias in neural dynamics, especially considering that EEG measures such as oscillatory power and functional connectivity can differ by sex. Such limitations arose due to the voluntary nature of participation, and further sample expansion was not feasible since February 2022. Additional recruitment under such conditions could confound results by introducing stress-related variability unrelated to the study’s focus. These factors should be considered when interpreting the results, and future studies should aim to include larger and more gender-balanced cohorts to enhance generalizability and statistical robustness.

EEG data were recorded throughout the stimulus presentation period at two different sites using two certified EEG systems. The first system—hardware complex “KhAI Medica” (Kharkiv, Ukraine)—was employed at Taras Shevchenko National University of Kyiv, while the second—“DX Systems” EEG system (Kharkiv, Ukraine)—was used in a hospital setting. Both systems are certified for clinical use in Ukraine and comply with national regulations for medical devices, including metrological standards for devices with measurement functions.

Although the EEG systems were from different manufacturers, they were functionally equivalent in terms of technical specifications: both featured the same number of channels, identical electrode layout based on the international 10–20% system, common reference to interconnected ear electrodes, and inter-electrode impedance kept below 5 kΩ. The sampling rate was 500 Hz across all channels. Additional matched parameters included amplifier bandwidth (0.1–100 Hz), electrode polarization voltage (<300 mV), signal range (0.5–8,000 μV), and power consumption (<2 W).

Following Ukrainian legislation, both EEG systems used in this study were subject to regulatory metrological control, ensuring reliable and consistent data quality. Such a setup aligns with standard practices in multicenter EEG studies and supports data comparability across different recording sites.

The study was approved by the Bioethics Committees of Luhansk State Medical University (Rivne, Ukraine) and Taras Shevchenko National University of Kyiv (Kyiv, Ukraine) protocol #1 issued on January 17, 2022, and written informed consent was obtained from each subject under the World Medical Association (WMA) Declaration of Helsinki—ethical principles for the medical research involving human subjects (Helsinki, Finland, June 1964), the Declaration of Principles on Tolerance (28th session of the General Conference of UNESCO, Paris, November 16, 1995), the Convention for the protection of Human Rights and Dignity of the Human Being with regard to the Application of Biology and Medicine: Convention on Human Rights and Biomedicine (Oviedo, April 04, 1997).

The stimuli presentation included three categories of olfactory samples: ammonia (10% solution, “Phytopharm,” Bakhmut, Ukraine; trigeminal odorant), isoamyl acetate (“UkrAroma,” Dnipro, Ukraine; olfactory odorant), and mountain pine (*p. mugo*) essential oil (“Aromatika,” Kyiv, Ukraine; mixed odorant). These three agents were selected because the scent samples were applied to the blotting paper pieces and placed into the tightly closed test tubes. According to the instructions, patients had to open the tube and find a position of the sample where they could report the appearance of the odor sensation and did not experience discomfort.

The stimuli were presented in a sequence with 30-s-long intervals between presentations. Each stimulus was presented for 60 s, and the first 10 s (stimulus detection and recognition) were taken into further analysis. The resting state (open eyes) data was recorded for 60 s, with the first 10 s taken into analysis. The obtained EEG data were further pre-processed using the EEGLAB toolbox (Brunner et al., 2013), where the main preprocessing steps were carried out. The preprocessing algorithm included raw data inspection, data filtering ([0.1 Hz; 30.1 Hz]), identification and rejection of bad channels with further interpolation, removal of artifactual time points, ICA analysis, and component rejection.

The EEG data were subdivided into the following oscillatory subbands, allowing the evaluation of the induced responses generated by the human cortex: 𝜃1 [3.5 5.9] Hz, 𝜃2 [6.0 7.4] Hz, 𝛼1 [7.5 9.4] Hz, 𝛼2 [9.5 10.7] Hz, 𝛼3 [10.8 13.5] Hz, β1 [13.6 19.9] Hz, β2 [20 30] Hz.

### PSD

The Power Spectral Density (PSD) analysis technique was applied to the recorded EEG data while processing various stimuli. The process involved computing the Fast Fourier Transform (FFT), squaring it to estimate the PSD, and then normalizing it within specified frequency ranges for each EEG channel. The contribution of power within each frequency range, referred to as the PSD value below, was calculated relative to the total spectral power. First, Welch’s method (Welch, 1967) was used to compute the PSD ${\hat{P}}_{\mathrm{ch}}(f)$ for each EEG channel ch . Next, the PSD was integrated over specific frequency bands to extract the power in each band. Finally, the total power normalized this band-specific power across the entire frequency range to yield the PSD value. Formally, for a given channel ch and a frequency band [f1,f2] , the normalized PSD is defined as:

$$\mathrm{PS}D_{\mathrm{ch}}(f1,f2)=\frac{\int_{f1}^{f2}{\hat{P}}_{\mathrm{ch}}(f)\mathrm{df}}{\int_{\text{fmin}}^{\text{fmax}}{\hat{P}}_{\mathrm{ch}}(f)\mathrm{df}}.$$

Here, ${\hat{P}}_{\mathrm{ch}}(f)$ is the channel-specific PSD estimate, obtained via the FFT and averaged under Welch’s method. The frequency limits $f_{\min}$ and $f_{\max}$ span the total frequency range of interest (e.g., 3.5–30 Hz for typical EEG band analyses). This ratio highlights each band’s relative contribution to the EEG signal’s total spectral power. Consequently, these PSA values enable a deeper examination of how different frequency bands respond to various stimuli (Chernykh et al., 2022).

### DMA of EEG band power modulation

The long-range correlation observed in EEG band power modulation was quantified by the detrended moving average (DMA) method (Tsujimoto et al., 2016). DMA is a powerful tool for estimating the scaling exponent of long-range correlated time series, including EEG data recorded during cognitive stimulus processing. The methodology for obtaining each frequency band power modulation includes several steps: First, the time series for each EEG channel is filtered using a Butterworth band-pass filter based on specified EEG bands. Then, the Hilbert transform (Feldman, 2011) is applied to each filtered signal to obtain the analytic signal, with the envelope estimated as the absolute value of the analytic signal (Chernykh et al., 2022).

The transformed signal is integrated in the following steps, and a fluctuation function of the integrated series is computed according to the common DMA procedure. This is done using the m-th order Savitzky–Golay smoothing filter (Savitzky and Golay, 1964), with a window length of *s* points. The power-law scaling range is determined by identifying a linear relationship on a double-logarithmic plot of the fluctuation function 𝑙𝑜𝑔(𝐹(𝑠)) against scales 𝑙𝑜𝑔(𝑠) (Chernykh et al., 2022).

The scaling exponent 𝛼 is derived from the slope of the linear part of this plot, providing critical insights into the signal:

0 < 𝛼 < 0.5 indicates a long-range anticorrelated signal;
𝛼 = 0 indicates an uncorrelated signal (white noise);
0.5 < 𝛼 < 1 denotes a long-range correlated signal;
𝛼 = 1 corresponds to 1/f-noise, also known as pink noise;
𝛼 > 1 implies a non-stationary, unbounded signal;
𝛼 = 3/2 corresponds to Brownian noise as integrated white noise.

Recent studies have shown that 1/f noise, or pink noise, is not a random background phenomenon observed in neural signals but is functionally linked to cognitive performance and information processing efficiency. In particular, 1/f spectral properties are considered indicative of self-organized criticality in neural networks, a regime that supports an optimal balance between stability and flexibility in brain dynamics. For example, Aguilar-Velázquez and Guzmán-Vargas (2019) demonstrated that rich-club network architectures with a balanced distribution of excitatory and inhibitory hub neurons can generate critical synchronization patterns characterized by 1/f-like spectra. Ouyang et al. (2020) reported that the 1/f component of resting-state EEG is independently associated with cognitive processing speed, and Waschke et al. (2021) found that 1/f neural noise correlates with activity in the noradrenergic system during cognitive control, suggesting a close link to attentional and executive functions.

These findings support the interpretation of 1/f characteristics as neural efficiency and cognitive readiness indicators. In this study, we use the scaling exponent 𝛼 obtained from the DMA method to quantify these dynamics. As noted above, 𝛼 = 1 corresponds to 1/f noise and is thus regarded as a marker of increased complexity in brain activity. It is important to note that while the observed *α*-values in certain brain regions align with patterns described in previous studies (e.g., Scarciglia et al., 2025; Wijnants, 2014), these associations are preliminary. The interpretations should be considered as hypotheses that require further empirical validation.

The results were statistically evaluated using the Mann–Whitney test for the intragroup analysis and the Kruskal-Wallis test for the intergroup comparisons, and *p* < 0.05 was considered significant. As all frequency bands analyzed in this work are derived from the same EEG recording, Bonferroni correction was applied to account for the multiple comparisons.

## Results

We provide here a condensed description of the main results; the extended description of the obtained results is given in Appendix.

### Resting state

At baseline, clear group-level distinctions were evident in EEG patterns. Individuals with a history of severe COVID-19 (Group S) showed abnormally high power in the upper beta range (notably β2) over temporo-parieto-occipital regions relative to both moderately affected (Group M) and healthy control (Group H) groups. Concurrently, Group S exhibited stronger long-range temporal correlations in low-frequency bands, reflected by elevated detrended moving average scaling exponents in the θ1–θ2 ranges, indicating more rigid, less complex neural signal dynamics than the other groups. In contrast, the moderate COVID-19 recovery group (M) showed a more typical or intermediate EEG profile. Group M’s power spectral density was generally low across most bands, aside from a focal enhancement of *α*_3-band power in posterior regions. This localized α activity in Group M may signify partial cortical reorganization, as it exceeded levels in both Group S and Group H.

Meanwhile, healthy controls (H) demonstrated the expected hallmarks of eyes-closed resting EEG – a dominant occipital α rhythm (maximal in the α_2 band) with broadly coherent dynamics. Their DMA measures indicated predominantly stationary, correlated oscillatory processes, aligning with normal flexible neurodynamics.

### Trigeminal stimulation (ammonia)

Upon exposure to the ammonia odor (a trigeminal stimulus), only the severe COVID group exhibited a notable reactivity in EEG measures. Specifically, Group S showed a significant increase in α_2-band power concentrated over midline and posterior cortical regions compared to its own resting baseline. This suggests that trigeminal input elicited an engagement of oscillatory activity around the 10 Hz range in the severely affected cohort. In contrast, no significant change in PSD occurred in Group M or Group H during ammonia stimulation. Likewise, DMA scaling exponents remained largely unchanged across all groups (including Group S), indicating that the fundamental temporal structure of EEG fluctuations was stable in the face of this stimulus. In summary, the trigeminal odor provoked a mild power augmentation in the severe group’s cortex (notably in the α_2 band). In contrast, the moderate and control groups showed no measurable EEG reactivity to the ammonia stimulus.

### Olfactory stimulation (isoamyl acetate)

Introduction of the isoamyl acetate odor (a pure olfactory stimulus) did not produce any statistically significant within-group changes in cortical EEG activity. None of the three groups exhibited a reliable shift in band power or DMA-based measures during the odor presentation relative to the resting state. Visual inspection had suggested slight increases in posterior α_2/α_3 activity in Group S and localized β_1 enhancements in Group M, but these did not reach significance when tested formally. Although the olfactory stimulus alone failed to drive acute EEG responses, notable differences between groups persisted under this condition. In inter-group comparisons, the severe COVID-19 group maintained significantly higher high-frequency power than the others – PSD in the β_1–β_2 range was globally elevated in Group S. Group S also showed broadly greater DMA scaling exponents in lower-frequency bands (especially α_1, α_2, θ_1, θ_2), reflecting an overall more autocorrelated (less dynamically flexible) signal across the cortex. In contrast, Group M’s EEG during isoamyl acetate showed only subtle changes (e.g., a slight focal increase in α_3 power over posterior regions), and Group H remained physiologically typical with no abnormal patterns. Thus, even in the absence of overt olfactory-driven reactivity, the severe group’s cortical activity stood out for its heightened fast-wave synchrony and sustained temporal correlations relative to the other groups.

### Pine scent stimulation

During the mixed pine scent stimulation (which engages both olfactory and trigeminal pathways), pronounced between-group differences emerged despite minimal intra-group changes. As with isoamyl acetate, no significant power or DMA alterations occurred from pre-stimulus baseline in any single group exposed to pine (all *p* > 0.05). However, the severe COVID-19 group continued to display abnormal hypersynchrony under this multisensory condition. Group S exhibited a dominant increase in β_2-band power, with significantly higher PSD in the beta range over broad central and posterior cortex areas compared to Groups M and H. At the same time, Group S showed widespread elevations in the DMA scaling exponent across most frequency bands (notably in α_1, α_2, θ_1, θ_2 subbands), consistent with heightened long-range autocorrelation and reduced signal complexity. By comparison, the moderate group again exhibited only limited changes – a modest focal increase in α_3 power over posterior regions was the main spectral difference for Group M, and a few isolated DMA differences appeared in frontal or temporal areas. Healthy participants showed no areas of topographic dominance in any band during pine stimulation, reflecting a uniformly normal response profile.

Together, these findings underscore a pattern of persistent cortical hypersynchrony and elevated temporal rigidity in individuals from Group S, in stark contrast to the more localized or negligible effects seen in Groups M and H. This group disparity was evident at rest and remained apparent even during sensory challenges, suggesting ongoing neurodynamic dysregulation in the post-severe COVID brain.

## Discussion

In the resting-state condition, statistical comparisons confirmed that Group S exhibited significantly elevated PSD values in the β1 and β2 bands, particularly in the bilateral temporo-parieto-occipital (TPO) regions, compared to both Group M and Group H (Supplementary Figures 1A, 4). DMA values in Group S were also significantly elevated in the θ1 and θ2 subbands, consistent with heightened temporal autocorrelation and reduced signal complexity (Supplementary Figures 1B, 4). These findings suggest maladaptive cortical dynamics that may underlie impaired executive function and mental fatigue (Lamontagne et al., 2021).

The presence of Brownian noise-like dynamics in frontal and occipital regions, along with suppressed α1 power (Everhart and Demaree, 2003), further reflected deficits in emotional regulation and signal stability. Collectively, these resting-state features point to disrupted integration across distributed cortical networks (Brooks et al., 2018).

In contrast, Group M showed significant increases in α3 power, particularly over the right posterior regions (Supplementary Figures 2A, 4), alongside stable DMA values in the θ1, α1, and α3 subbands. These findings are compatible with neural reorganization and suggest partial re-engagement of frontoparietal and memory networks (Sadaghiani et al., 2012, 2019; Brooks et al., 2018). However, elevated α3 activity has also been associated with mild cognitive impairment (Moretti, 2015a, 2015b, 2018), suggesting that such reorganization may coexist with residual dysfunction.

Group H exhibited typical resting-state neurodynamics, with strong α2 power localized to occipital and frontal regions (Supplementary Figures 3A, 4), reflecting cognitive readiness (Fuentes-Claramonte et al., 2019; Snipes et al., 2022). DMA results indicated stable signal coherence and dynamic switching, although some variability in right-hemispheric and temporal zones was noted (Sauseng et al., 2010; Biel et al., 2021).

During ammonia (trigeminal) stimulation, Group S exhibited a statistically significant increase in α2 PSD over midline and posterior cortical areas, while θ1, θ2, and α2 subbands also showed elevated DMA values (Supplementary Figures 8A, 9A, 10). These changes suggest increased signal rigidity and sustained cortical excitation under sensory load. Neither Group M nor Group H demonstrated significant within-group changes during ammonia exposure (Supplementary Figures 8B,C, 9B,C), although Group H maintained focal α2 power and coherent θ1 and α3 DMA organization.

Under isoamyl acetate stimulation, no significant within-group changes were observed in any cohort (Supplementary Figures 14, 15). However, Group S showed dominant intergroup elevations in *β*1 and β2 PSD and broadly increased DMA values in α1, α2, θ1, and θ2 subbands (Supplementary Figure 16), reflecting pervasive signal overactivation and decreased neurodynamic flexibility. Group M exhibited localized α3 enhancements, while Group H showed minimal reactivity.

For the mixed (olfactory-trigeminal) pine scent, intragroup comparisons again showed no significant changes (Supplementary Figures 20, 21), but intergroup analysis revealed prominent increases in β2 PSD and widespread elevation of DMA exponents in Group S (Supplementary Figure 22), particularly across the *α*1–θ2 range. Group M showed *α*3 increases and focal β1/θ1 DMA elevations, whereas Group H demonstrated no dominant topographies.

Together, these results demonstrate that Group S is marked by hyperexcitable yet rigid cortical dynamics, with sustained β-band overactivation and elevated long-range temporal structuring across conditions. Group M exhibited partially recovered α3-linked memory-attentional patterns, consistent with partial reactivation of frontoparietal control systems (LaRocque et al., 2014; Jawabri and Cascella, 2020), while Group H maintained physiologically normative neurodynamics with minimal odorant-related modulation. The subtle modulations seen in Group H may also relate to transient shifts in functional coupling, such as those described during theta-gamma interaction and semantic encoding (Meyer et al., 2015; Kalafatovich et al., 2022). Structural interpretations—such as the role of the temporoparietal junction and orbitofrontal cortex in sensory integration (De Benedictis et al., 2014; Ogawa and Kameda, 2019)—may provide further avenues for contextualizing the observed neurodynamics.

Although pink (α ≈ 1) and Brownian (α ≈ 3/2) noise-like patterns suggest distinctive signal organization regimes, these findings should be interpreted cautiously in the absence of behavioral data. Nonetheless, their interpretation aligns with prior research linking pink noise to cognitive efficiency and Brownian dynamics to neural instability (Aguilar-Velázquez and Guzmán-Vargas, 2019; Ouyang et al., 2020; Waschke et al., 2021).

Our results support the use of DMA as a complementary metric to PSD for quantifying temporal structure in EEG signals and highlight the potential of EEG-based neurodynamic biomarkers for understanding post-COVID cortical dysregulation.

Future studies combining electrophysiological and behavioral data will be essential to validate these findings and clarify the role of long-range signal autocorrelation in cognitive recovery.

While the current study emphasized robust statistical effects, future research might revisit the role of more nuanced, stimulus-specific or topographically localized effects—especially in Group M and H—through the lens of prior functional imaging and electrophysiological findings. For instance, evidence of alpha-band phase synchrony within frontoparietal networks (Sadaghiani et al., 2012) or dynamic reconfiguration of theta-beta coupling during memory retrieval (Meyer et al., 2015; Kalafatovich et al., 2022) could help contextualize subtle trends that did not survive correction. Furthermore, the anatomical-functional connectivity across TPO and orbitofrontal regions described in neurosurgical studies (De Benedictis et al., 2014) or the differential roles of left and right TPJ in competitive interactions (Ogawa and Kameda, 2019) might offer a structural rationale for future neurodynamic research integrating multimodal imaging, EEG, and behavioral measures.

The interpretations of observed pink noise (*α* ≈ 1) and Brownian noise (α ≈ 3/2) patterns provide intriguing insights into neural dynamics; we acknowledge that these conclusions remain tentative without direct behavioral or neuropsychological assessments. In this study, we did not collect concurrent cognitive or task-based performance data due to the constraints imposed by clinical environments during the COVID-19 pandemic.

However, our interpretation of these noise characteristics is informed by prior research. Pink noise has been associated with optimal information integration, self-organized criticality, and adaptive flexibility in cognitive processing (Aguilar-Velázquez and Guzmán-Vargas, 2019; Ouyang et al., 2020; Waschke et al., 2021). Conversely, Brownian noise has been linked to highly non-stationary processes that may reflect impaired or unstructured neural information flow.

Thus, classifying the α-scaling exponent into these categories provides a useful theoretical framework for characterizing the large-scale dynamics of the brain under pathological and recovery conditions. Our findings contribute to this framework by demonstrating how the distribution of α values across cortical regions varies between patients with severe, moderate, and no history of COVID-19.

Nevertheless, we emphasize that these interpretations should be viewed as hypothesis-generating rather than conclusive, and future studies incorporating behavioral or neurocognitive assessments will be essential for validating and extending these observations.

## Conclusion

This study revealed distinct cortical neurodynamic patterns in response to resting and olfactory-trigeminal stimulation among individuals with varying COVID-19 histories. Group S consistently demonstrated abnormal EEG patterns characterized by elevated high-frequency spectral power, global increases in DMA scaling exponents, and poor stimulus-related modulation. These findings suggest hyperexcitable and temporally rigid cortical activity, potentially reflecting persistent dysregulation of large-scale networks. However, in the absence of direct neuropsychological or behavioral data, such interpretations must be viewed as exploratory and hypothesis-generating rather than confirmatory.

Group M exhibited electrophysiological evidence of partial functional reorganization, including *α*3-associated increases and stable coherence patterns in key frequency bands, although without statistically significant within-group reactivity to olfactory stimuli. These results may reflect limited but present neuroplastic adaptations during the post-COVID recovery phase.

Healthy controls (Group H) displayed canonical resting-state EEG features and minimal reactivity to olfactory cues, with localized coherence structure changes interpreted as typical physiological modulation.

The combined use of PSD and DMA analyses revealed complementary aspects of brain dynamics—amplitude-based and temporal-structural—that jointly offer a more nuanced perspective on potential cortical changes following COVID-19. In particular, altered α-scaling exponent distributions observed in Group S may serve as candidate EEG-based indicators of atypical signal organization. Nevertheless, without concurrent neuropsychological testing, interpretations related to cognitive dysfunction or recovery remain speculative.

These findings highlight the importance of future longitudinal studies integrating electrophysiological, neurocognitive, and behavioral assessments to validate EEG-derived biomarkers and guide post-COVID monitoring and rehabilitation efforts.

This study revealed distinct cortical neurodynamic patterns in response to resting and olfactory-trigeminal stimulation among individuals with varying COVID-19 histories. Group S consistently demonstrated abnormal EEG patterns characterized by elevated high-frequency spectral power, global increases in DMA scaling exponents, and poor stimulus-related modulation. These findings suggest hyperexcitable and temporally rigid cortical activity, reflecting persistent dysregulation even during sensory processing.

Group M exhibited electrophysiological evidence of partial functional recovery, including *α*3-associated increases and stable coherence patterns in key frequency bands, but did not show statistically significant within-group reactivity to olfactory stimuli. This may indicate constrained but present neuroplastic adaptations during the post-COVID recovery phase.

Healthy controls (Group H) displayed canonical resting-state patterns and minimal reactivity to olfactory cues, with localized changes in coherence structure that reflect flexible and efficient cortical functioning.

The combined use of PSD and DMA analyses highlighted complementary aspects of neurodynamics—amplitude-based and temporal-correlational—that jointly offer a more holistic perspective on the cortical aftermath of COVID-19. The observed elevations in α-scaling exponents in Group S suggest that temporal dynamics could serve as a useful biomarker of cognitive vulnerability or delayed recovery.

These findings underscore the need for longitudinal studies incorporating behavioral and cognitive assessments to validate electrophysiological biomarkers and inform targeted rehabilitation strategies.

Future research may further investigate and validate the detrended moving average (DMA) method for assessing the state of neural networks based on EEG data to enhance its predictive utility and generalizability. Another promising direction involves examining the long-term effects of COVID-19, in conjunction with other significant stressors, such as those related to armed conflict, on brain function.

Among the limitations of the present study is the imbalance in the sample composition, which arose from the voluntary nature of participant recruitment. Furthermore, potential confounding effects related to regional differences in the participants’ social and environmental backgrounds should be considered, as individuals in Group S were residents of Sievierodonetsk. In contrast, participants in Groups M and H were recruited from Kyiv.

## Data Availability

The raw data supporting the conclusions of this article will be made available by the authors, without undue reservation.

## References

[ref1] Aguilar-Velázquez D. Guzmán-Vargas L. (2019). Critical synchronization and 1/f noise in inhibitory/excitatory rich-club neural networks. Sci. Rep. 9:1258. doi: 10.1038/s41598-018-37920-w, PMID: 30718817 PMC6361933

[ref2] Antony A. R. Haneef Z. (2020). Systematic review of EEG findings in 617 patients diagnosed with COVID-19. Seizure 83, 234–241. doi: 10.1016/j.seizure.2020.10.014, PMID: 33121875 PMC7569418

[ref6] Biel A. L. Minarik T. Sauseng P. (2021). EEG cross-frequency phase synchronization as an index of memory matching in visual search. NeuroImage 235:117971. doi: 10.1016/j.neuroimage.2021.117971, PMID: 33839263

[ref8] Brooks J. R. Passaro A. D. Kerick S. E. Garcia J. O. Franaszczuk P. J. Vettel J. M. (2018). Overlapping brain network and alpha power changes suggest visuospatial attention effects on driving performance. Behav. Neurosci. 132, 23–33. doi: 10.1037/bne0000224, PMID: 29389145

[ref9] Brunner C. Delorme A. Makeig S. (2013). Eeglab – an open source matlab toolbox for electrophysiological research. Biomed. Tech 58 Suppl 1. doi: 10.1515/bmt-2013-4182, PMID: 24042816

[ref10] Carbone A. Kiyono K. (2016). Detrending moving average algorithm: frequency response and scaling performances. Phys. Rev. E 93:063309. doi: 10.1103/physreve.93.063309, PMID: 27415389

[ref11] Ceban F. Ling S. Lui L. M. W. Lee Y. Gill H. Teopiz K. M.. (2022). Fatigue and cognitive impairment in post-COVID-19 syndrome: a systematic review and meta-analysis. Brain Behav. Immun. 101, 93–135. doi: 10.1016/j.bbi.2021.12.020, PMID: 34973396 PMC8715665

[ref12] Chaput M. A. El Mountassir F. Atanasova B. Thomas-Danguin T. Le Bon A. M. Perrut A.. (2012). Interactions of odorants with olfactory receptors and receptor neurons match the perceptual dynamics observed for woody and fruity odorant mixtures. Eur. J. Neurosci. 35, 584–597. doi: 10.1111/j.1460-9568.2011.07976.x, PMID: 22304504

[ref13] Chehrehnegar N. Nejati V. Shati M. Rashedi V. Lotfi M. Adelirad F.. (2019). Early detection of cognitive disturbances in mild cognitive impairment: a systematic review of observational studies. Psychogeriatrics 20, 212–228. doi: 10.1111/psyg.12484, PMID: 31808989

[ref14] Chernykh M. Vodianyk B. Seleznov I. Harmatiuk D. Zyma I. Popov A.. (2022). Detrending moving average, power spectral density, and coherence: three eeg-based methods to assess emotion irradiation during facial perception. Appl. Sci. 12:7849. doi: 10.3390/app12157849

[ref16] De Benedictis A. Duffau H. Paradiso B. Grandi E. Balbi S. Granieri E.. (2014). Anatomo-functional study of the temporo-parieto-occipital region: dissection, tractographic and brain mapping evidence from a neurosurgical perspective. J. Anat. 225, 132–151. doi: 10.1111/joa.12204, PMID: 24975421 PMC4111924

[ref17] Devanand D. P. Liu X. Tabert M. H. Pradhaban G. Cuasay K. Bell K.. (2008). Combining early markers strongly predicts conversion from mild cognitive impairment to Alzheimer's disease. Biol. Psychiatry 64, 871–879. doi: 10.1016/j.biopsych.2008.06.020, PMID: 18723162 PMC2613777

[ref18] Díez-Cirarda M. Yus M. Gómez-Ruiz N. Polidura C. Gil-Martínez L. Delgado-Alonso C.. (2022). Multimodal neuroimaging in post-COVID syndrome and correlation with cognition. Brain 146, 2142–2152. doi: 10.1093/brain/awac384, PMID: 36288544 PMC9620345

[ref19] Everhart D. E. Demaree H. A. (2003). Low alpha power (7.5-9.5 Hz) changes during positive and negative affective learning. Cogn. Affect. Behav. Neurosci. 3, 39–45. doi: 10.3758/cabn.3.1.39, PMID: 12822597

[ref20] Feldman M. (2011). Hilbert transform in vibration analysis. Mech. Syst. Signal Process. 25, 735–802. doi: 10.1016/j.ymssp.2010.07.018

[ref21] Fuentes-Claramonte P. Martín-Subero M. Salgado-Pineda P. Alonso-Lana S. Moreno-Alcázar A. Argila-Plaza I.. (2019). Shared and differential default-mode related patterns of activity in an autobiographical, a self-referential and an attentional task. PLoS One 14:e0209376. doi: 10.1371/journal.pone.0209376, PMID: 30608970 PMC6319771

[ref22] Fung T. K. H. Lau B. W. M. Ngai S. P. C. Tsang H. W. H. (2021). Therapeutic effect and mechanisms of essential oils in mood disorders: interaction between the nervous and respiratory systems. Int. J. Mol. Sci. 22:4844. doi: 10.3390/ijms22094844, PMID: 34063646 PMC8125361

[ref23] Gu H. Yao Q. Chen H. Ding Z. Zhao X. Liu H.. (2022). The effect of mental schema evolution on mental workload measurement: an EEG study with simulated quadrotor UAV operation. J. Neural Eng. 19:026058. doi: 10.1088/1741-2552/ac6828, PMID: 35439750

[ref24] Gu G.-F. Zhou W.-X. (2010). Detrending moving average algorithm for multifractals. Phys. Rev. E 82:011136. doi: 10.1103/physreve.82.011136, PMID: 20866594

[ref25] Hampshire A. Trender W. Chamberlain S. R. Jolly A. E. Grant J. E. Patrick F.. (2021). Cognitive deficits in people who have recovered from COVID-19. EClinicalMedicine 39:101044. doi: 10.1016/j.eclinm.2021.101044, PMID: 34316551 PMC8298139

[ref26] Herman C. Mayer K. Sarwal A. (2020). Scoping review of prevalence of neurologic comorbidities in patients hospitalized for COVID-19. Neurology 95, 77–84. doi: 10.1212/wnl.0000000000009673, PMID: 32345728

[ref27] Hwang S. T. Ballout A. A. Sonti A. N. Kapyur A. Kirsch C. Singh N.. (2021). EEG abnormalities and their radiographic correlates in a COVID-19 inpatient cohort. Neurol. Clin. Pract. 12, 52–59. doi: 10.1212/cpj.0000000000001136, PMID: 36157621 PMC9491502

[ref28] Jawabri K. Cascella M. (2020) Physiology, explicit memory – statpearls – NCBI bookshelf. *National Center for Biotechnology Information*. Available online at: https://www.ncbi.nlm.nih.gov/books/NBK554551/

[ref29] Kalafatovich J. Lee M. Lee S.-W. (2022). Decoding declarative memory process for predicting memory retrieval based on source localization. PLoS One 17:e0274101. doi: 10.1371/journal.pone.0274101, PMID: 36074790 PMC9455842

[ref31] Lamontagne S. J. Winters M. F. Pizzagalli D. A. Olmstead M. C. (2021). Post-acute sequelae of COVID-19: evidence of mood & cognitive impairment. Brain Behav. Immun. Health 17:100347. doi: 10.1016/j.bbih.2021.100347, PMID: 34549199 PMC8437695

[ref32] LaRocque J. J. Lewis-Peacock J. A. Postle B. R. (2014). Multiple neural states of representation in short-term memory? It’s a matter of attention. Front. Hum. Neurosci. 8:5. doi: 10.3389/fnhum.2014.00005, PMID: 24478671 PMC3899521

[ref33] Leonel Tavares-Júnior J. W. Caetano de Souza A. C. Pereira Borges J. W. Oliveira D. N. Siqueira-Neto J. I. Sobreira-Neto M. A. (2022). Covid-19 associated cognitive impairment: a systematic review. Cortex 152, 77–97. doi: 10.1016/j.cortex.2022.04.006, PMID: 35537236 PMC9014565

[ref34] Méndez R. Balanzá-Martínez V. Luperdi S. C. Estrada I. Latorre A. González-Jiménez P.. (2021). Short-term neuropsychiatric outcomes and quality of life in COVID-19 survivors. J. Intern. Med. 290, 621–631. doi: 10.1111/joim.13262, PMID: 33533521 PMC8013333

[ref35] Meyer L. Grigutsch M. Schmuck N. Gaston P. Friederici A. D. (2015). Frontal–posterior theta oscillations reflect memory retrieval during sentence comprehension. Cortex 71, 205–218. doi: 10.1016/j.cortex.2015.06.027, PMID: 26233521

[ref37] Moretti D. V. (2015a). Conversion of mild cognitive impairment patients in Alzheimer’s disease: prognostic value of Alpha3/Alpha2 electroencephalographic rhythms power ratio. Alzheimers Res. Ther. 7:80. doi: 10.1186/s13195-015-0162-x, PMID: 26715588 PMC4696332

[ref38] Moretti V. D. (2015b). Atrophy and lower regional perfusion of temporo-parietal brain areas are correlated with impairment in memory performances and increase of EEG upper alpha power in prodromal Alzheimer's disease. Am. J. Neurodegener. Dis. 4, 13–27, PMID: 26389016 PMC4568770

[ref39] Moretti D. V. (2018). Increase of EEG alpha3/alpha2 power ratio detects inferior parietal lobule atrophy in mild cognitive impairment. Curr. Alzheimer Res. 15, 443–451. doi: 10.2174/1567205014666171030105338, PMID: 29086693

[ref40] Nath A. (2020). Neurologic complications of coronavirus infections. Neurology 94, 809–810. doi: 10.1212/wnl.0000000000009455, PMID: 32229625

[ref41] Ogawa A. Kameda T. (2019). Dissociable roles of left and right temporoparietal junction in strategic competitive interaction. Soc. Cogn. Affect. Neurosci. 14, 1037–1048. doi: 10.1093/scan/nsz082, PMID: 31680151 PMC6970153

[ref42] Ouyang G. Hildebrandt A. Schmitz F. Herrmann C. S. (2020). Decomposing alpha and 1/f brain activities reveals their differential associations with cognitive processing speed. NeuroImage 205:116304. doi: 10.1016/j.neuroimage.2019.116304, PMID: 31654760

[ref43] Paolini M. Palladini M. Mazza M. G. Colombo F. Vai B. Rovere-Querini P.. (2022). Brain correlates of subjective cognitive complaints in COVID-19 survivors: a multimodal magnetic resonance imaging study. Eur. Neuropsychopharmacol. 68, 1–10. doi: 10.1016/j.euroneuro.2022.12.002, PMID: 36640728 PMC9742225

[ref44] Petrova M. Diamond J. Schuster B. Dalton P. (2008). Evaluation of trigeminal sensitivity to ammonia in asthmatics and healthy human volunteers. Inhal. Toxicol. 20, 1085–1092. doi: 10.1080/08958370802120396, PMID: 18728993 PMC2737692

[ref45] Poletti S. Palladini M. Mazza M. G. De Lorenzo R. Furlan R. Ciceri F.. (2021). Long-term consequences of COVID-19 on cognitive functioning up to 6 months after discharge: role of depression and impact on quality of life. Eur. Arch. Psychiatry Clin. Neurosci. 272, 773–782. doi: 10.1007/s00406-021-01346-9, PMID: 34698871 PMC8546751

[ref9001] Sadaghiani S. Scheeringa R. Lehongre K. Morillon B. Giraud A. L. Giraud A. L. (2012). α-band phase synchrony is related to activity in the fronto-parietal adaptive control network. The Journal of Neuroscience, 32, 14305–14310. doi: 10.1523/JNEUROSCI.1358-12.2012 23055501 PMC4057938

[ref9002] Sadaghiani S. Dombert P. L. Løvstad M. Funderud I. Meling T. R. Endestad T.. (2019). Lesions to the Fronto-Parietal Network Impact Alpha-Band Phase Synchrony and Cognitive Control. Cerebral Cortex, 29, 4143–4153. doi: 10.1093/cercor/bhy296 30535068 PMC6735251

[ref48] Sauseng P. Griesmayr B. Freunberger R. Klimesch W. (2010). Control mechanisms in working memory: a possible function of EEG theta oscillations. Neurosci. Biobehav. Rev. 34, 1015–1022. doi: 10.1016/j.neubiorev.2009.12.006, PMID: 20006645

[ref49] Savitzky A. Golay M. J. E. (1964). Smoothing and differentiation of data by simplified least squares procedures. Anal. Chem. 36, 1627–1639. doi: 10.1021/ac60214a047 22324618

[ref50] Scarciglia A. Bonanno C. Valenza G. (2025). Physiological noise: a comprehensive review on informative randomness in neural systems. Phys Life Rev 53, 281–293. doi: 10.1016/j.plrev.2025.04.001, PMID: 40245660

[ref51] Seleznov I. Zyma I. Kiyono K. Tukaev S. Popov A. Chernykh M.. (2019). Detrended fluctuation, coherence, and spectral power analysis of activation rearrangement in EEG dynamics during cognitive workload. Front. Hum. Neurosci. 13:270. doi: 10.3389/fnhum.2019.00270, PMID: 31440151 PMC6694837

[ref52] Snipes S. Krugliakova E. Meier E. Huber R. (2022). The theta paradox: 4-8 Hz EEG oscillations reflect both sleep pressure and cognitive control. J. Neurosci. 42, 8569–8586. doi: 10.1523/jneurosci.1063-22.2022, PMID: 36202618 PMC9665934

[ref53] Thye A. Y.-K. Law J. W.-F. Tan L. T.-H. Pusparajah P. Ser H.-L. Thurairajasingam S.. (2022). Psychological symptoms in COVID-19 patients: insights into pathophysiology and risk factors of long COVID-19. Biology 11:61. doi: 10.3390/biology11010061, PMID: 35053059 PMC8773222

[ref54] Tsujimoto Y. Miki Y. Shimatani S. Kiyono K. (2016). Fast algorithm for scaling analysis with higher-order detrending moving average method. Phys. Rev. E 93:053304. doi: 10.1103/physreve.93.053304, PMID: 27301002

[ref55] Uchida S. Shimada C. Sakuma N. Kagitani F. Kan A. Awata S. (2020). The relationship between olfaction and cognitive function in the elderly. J. Physiol. Sci. 70:48. doi: 10.1186/s12576-020-00777-8, PMID: 33054707 PMC10717970

[ref56] Waschke L. Kloosterman N. A. Obleser J. Garrett D. D. (2021). Behavior needs neural variability. Neuron 109, 751–766. doi: 10.1016/j.neuron.2021.01.023, PMID: 33596406

[ref57] Welch P. (1967). The use of fast Fourier transform for the estimation of power spectra: a method based on time averaging over short, modified periodograms. IEEE Trans. Audio Electroacoust. 15, 70–73. doi: 10.1109/TAU.1967.1161901

[ref58] Wijnants M. L. (2014). A review of theoretical perspectives in cognitive science on the presence of 1/f scaling in coordinated physiological and cognitive processes. J. Nonlinear Dyn.:Article ID 962043. doi: 10.1155/2014/962043

