# Supplementary material for: Comparative EEG study of neurodynamics upon olfactory stimulation in COVID-19 patients
Source: Front Hum Neurosci. 2025 Jun 18;19:1571477. doi: 10.3389/fnhum.2025.1571477 (PMC12213800; doi:10.3389/fnhum.2025.1571477)
Supplement: Supplementary file 11 [file Supplementary_file_1.docx]

**Appendix**

**1.** **Resting State**

The analysis of the EEG data obtained during the examination revealed specific differences in the cortical oscillatory patterns in patients of the group participants, already during the resting state before the onset of sensory activation. Thus, Group S demonstrated a significant increase of activity within the
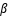
1 subband in both hemispheres in the temporal-occipital areas, with a pronounced maximum in the right posterior-temporal-parietal area (TPO). At the same time, it should be noted that the PSD value of other subbands (especially α2,
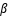
2,
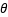
2, and α3) was close to zero normalized units (N.U.) (Supplementary Figure 1). In addition, the characteristics of the EEG signal, estimated using DMA, appeared to be non-stationary (α > 1) in the low-frequency part of the spectrum (
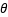
1,
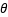
2, α1, α2, α3) and vice versa, correlated, coherent in the
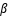
1,
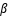
2 subbands (α > 1/2 ) (Supplementary Figure 1) generally across the cortex. The only exceptions were left temporal (T3), central (Cz), and right occipital (O2) leads, where the processes can be characterized as a state of "Brownian noise" (α=3/2) in the α1 subband of the EEG (Supplementary Figure 1).

**
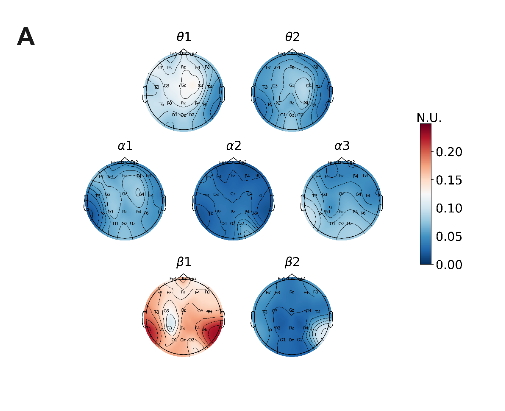
**
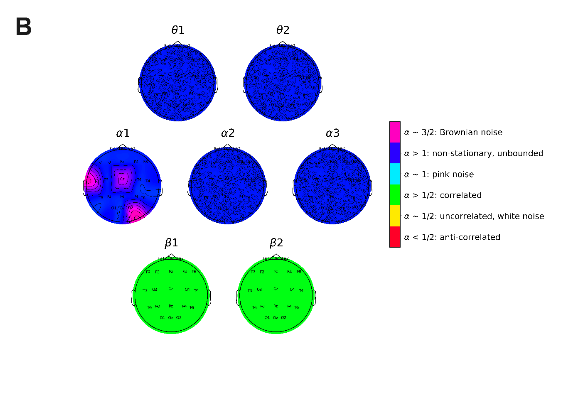


**Supplementary Figure 1. Topographical distribution of (A) PSD values, (B) α scaling exponent of DMA, calculated for the EEG data of the patients with severe course of COVID-19 disease (Group S, n=20) during resting state with closed eyes. Each scalp map corresponds to one EEG frequency subband: θ1, θ2, α1, α2, α3, β1, and β2. The color gradient represents (A) the values of the PSD coefficient and (B) the magnitude of the DMA scaling exponent.**

A completely different map of topographical distribution was observed in the resting state EEG data obtained from the group of subjects (Group M) who had COVID-19 and recovered (i.e., demonstrated negative PCR test results) 1-3 months prior to the experiment. The EEG of the Group M patients during the resting state was also characterized by the low level of oscillatory power in the
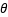
2 and
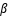
2 subbands, as well as reduced PSD values in the
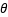
1, α1 and α2 subbands in almost all leads (Supplementary Figure 2). However, at the same time, the formation of a locus of enhanced power was detected in the right parietal-occipital area (Р4-О2-О1) in α3 subband, propagating the increased activity to the entire left hemisphere (Supplementary Figure 2). Withal, a local focus of minimum PSD values (<0.1 N.U.) was observed in the posterior temporal (T6) region.

**
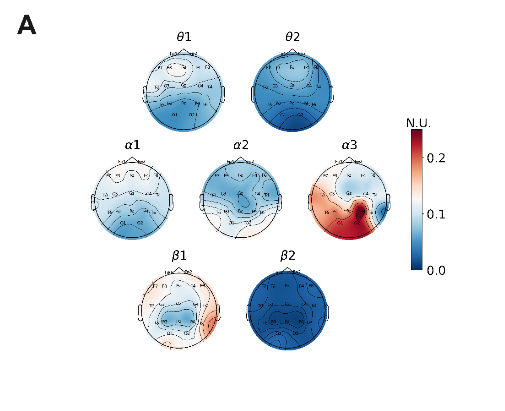
**

**
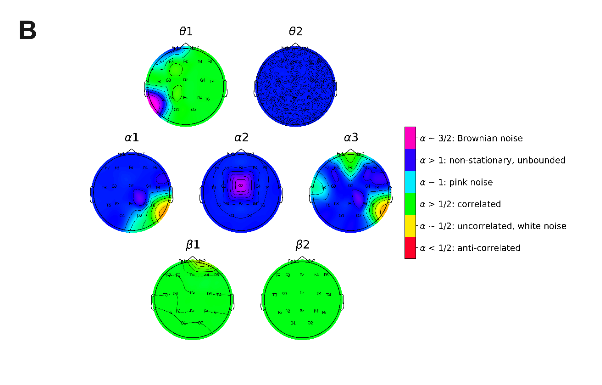
**

**Supplementary Figure 2. Topographical distribution of (A) PSD values and (B) α scaling exponent of DMA calculated for the EEG data of the patients with a moderate course of COVID-19 disease (Group M, n=21) during the resting state with closed eyes. Each scalp map corresponds to one EEG frequency subband: θ1, θ2, α1, α2, α3, β1, and β2. The color gradient represents (A) the values of the PSD coefficient and (B) the magnitude of the DMA scaling exponent.**

In addition, within the
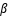
1 subband, such significant disturbances in the intensity of local synchronization processes were no longer observed, as in the case of resting state EEG of the Group S; however, a moderate increase in PSD (0.18 N.U.) was still detected, mainly in the temporal region of the right hemisphere (Supplementary Figure 2). It should also be noted that the use of the DMA made it possible to reveal the following: in the
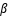
1,
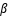
2,
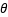
1 subbands of the EEG data were characterized as correlated, coherent (α > 1/2). In the
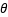
1 subband in the left hemispheric frontal (F3-F7) and temporal-parietal (T5-P3) areas, as non-stationary (α > 1) and Brownian noise (T5). In the remaining EEG bands, the dominance of non-stationary processes was observed, except for the right posterior temporal area (T6) in the α1, α3 subbands, where the properties of the signal tended to anticorrelation (α < ½); in the α3 subband within the anterior-central (F3-F4-Fz) and the left temporal region (T3) - the signals had a correlated structure (α > ½). In α2 subbands within the central area, a coefficient corresponded to Brownian noise (α =3/2) (Supplementary Figure 2).

**
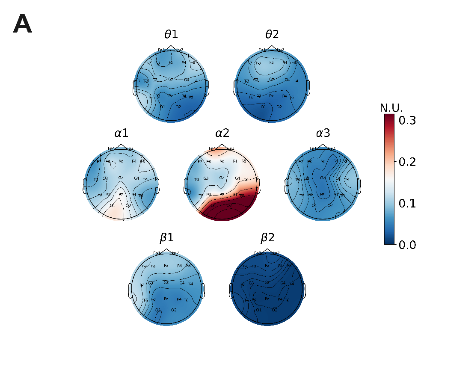
**

**
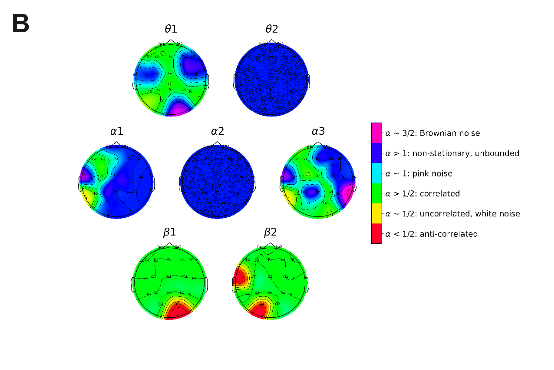
**

**Supplementary Figure 3. Topographical distribution of (A) PSD values, (B) α scaling exponent of DMA, calculated for the EEG data of the healthy control group (Group H, n=10) during the resting state with closed eyes. Each scalp map corresponds to one EEG frequency subband: θ1, θ2, α1, α2, α3, β1, and β2. The color gradient represents (A) the values of the PSD coefficient and (B) the magnitude of the DMA scaling exponents.**

The EEG patterns of the patients from the healthy control group (Group H), who have never been diagnosed with COVID-19, although experienced quarantine conditions and associated restrictions, were characterized by a classic power distribution, which corresponded to the generally accepted views on the resting state conditions: the prevalence of the α-rhythm (more specifically, α2 subband) with a maximum in the occipital and parietal areas, right TPO (> 0.3 N.U) and left frontal zone (~0.2 N.U.) was observed (Supplementary Figure 3). In addition, there was a slight increase in PSD values in the left occipital-parietal region in the α1 subband (~0.1 N.U.). Changes in PSD values in the remaining EEG subbands were low (up to 0.1 N.U.), especially in
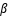
2 (< 0.01 N.U.).

The application of the DMA showed that the “cognitive” EEG subbands, i.e.,
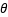
1, α3,
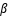
1, and
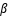
2 subbands, were characterized by the predominance of stationary coherent processes (α > ½). Yet several exceptions were detected: the
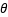
1 subband oscillations tended to non-stationarity in the left temporal, right frontal temporal zones (α >1), and the TPO area of the right hemisphere demonstrated transitions from pink to Brownian noise; the EEG signal in the
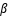
1 subband within the right TPO region demonstrated the anticorrelated structure. Within the
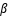
2 subband oscillations in the left temporal and occipital areas, levels of a-coefficient corresponded to anticorrelation structure levels (α < ½) (Supplementary Figure 3). Both
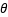
2, α2 subbands were characterized by total non-stationarity of oscillations in all leads (α > 1). At the same time, while α1 and α3 subbands in the right hemisphere were characterized by the presence of antistationarity in the development of fluctuation processes, the left hemisphere tended to correlation and coherence, except for T3- (left anterior temporal area; α~3/2; α>1 - Brownian noise combined with nonstationarity) and P3-channels (left anterior parietal area; α>1- non-stationary oscillations) in the α3 subband.

Supplementary Figure 4 represents the intergroup comparison of the PSD and DMA results obtained for the resting state EEG data. It can be noted that Group S demonstrated significantly elevated PSD values in the β2 subband in bilateral TPO regions compared to both Group M and Group H. This increase was not paralleled in other bands, which failed to survive correction. At the same time, group H demonstrated a widespread region of high PSD values within the right hemisphere for precentral and temporal regions and bilaterally within frontal and occipital zones. Group M demonstrated the highest values in the left parietal region. In DMA analysis, θ1 and θ2 subbands remained significantly elevated in Group S across the cortex, indicating long-range temporal dependencies. DMA values in α1, α2 subbands in the Group S demonstrated right-lateralized general increase and bilateral temporal-central loci of high DMA values, respectively.
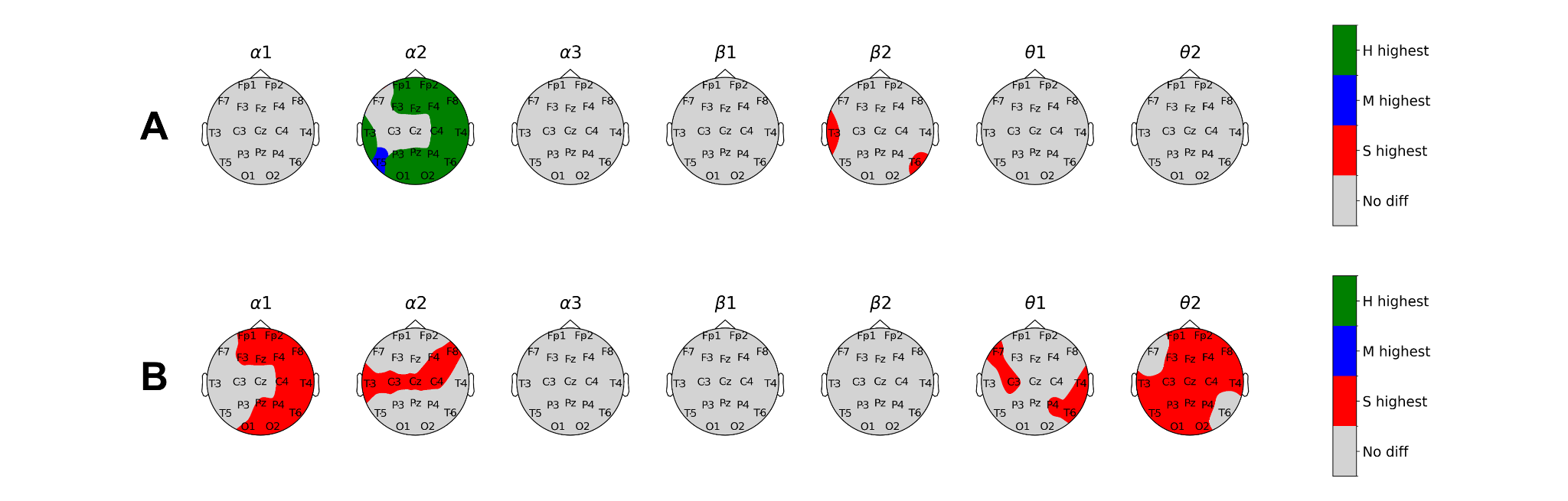


**Supplementary Figure 4. The topographical distribution of statistically significant differences in EEG power spectral density (PSD; Panel A) and detrended moving average (DMA; Panel B) across the three groups: individuals with severe COVID-19 (S), moderate COVID-19 (M), and healthy controls (H)—during the resting state condition. The data were analyzed using the non-parametric Kruskal–Wallis H test. Each subplot represents one EEG subband: α1, α2, α3, β1, β2, θ1, and θ2. The colored regions on each scalp topography indicate the group showing the highest value for that frequency band and electrode. The legend (right) defines the color scheme: green (healthy controls (H), highest), blue (moderate COVID-19 group (M), highest), red (severe COVID-19 group (S), highest), Gray (no significant differences), *p* < 0.05**

**2.** **Ammonia**

Activation of trigeminal receptors in patients of Group S was accompanied by a relative increase in PSD values in the
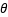
1, α3,
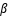
1 EEG subbands (Supplementary Figure 5). At the same time, the foci of maximum PSD values were observed in the temporal areas bilaterally with the relative dominance of right-sided structures. Changes in PSD value in
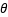
2, α1, α2, α3, and
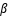
2 subranges were generally low across the cortex.


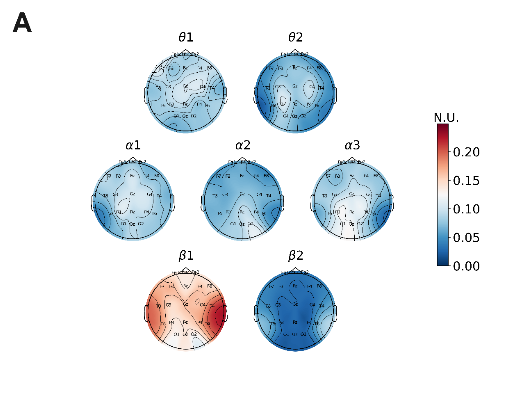


**
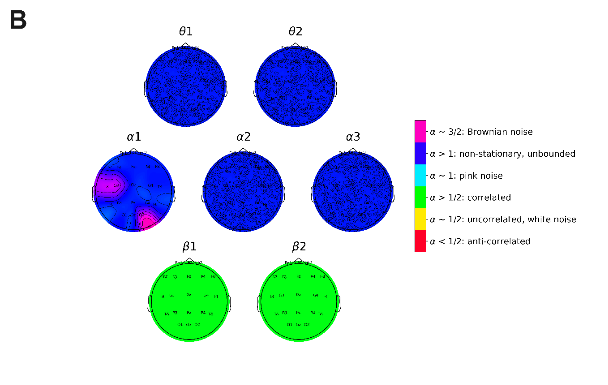
**

**Supplementary Figure 5. Topographical distribution of (A) PSD values, (B) α scaling exponent of DMA, calculated for the EEG data of the patients with severe course of COVID-19 disease (Group S, n=20) during exposure to ammonia smell sample (trigeminal stimulus). Each scalp map corresponds to one EEG frequency subband: θ1, θ2, α1, α2, α3, β1, and β2. The color gradient represents (A) the values of the PSD coefficient and (B) the magnitude of the DMA scaling exponent.**

The analysis of the EEG structure using the DMA in this group showed that perception of ammonia by Group S patients was characterized by a correlated (coherent) structure (α > 1/2) of the EEG fluctuations in the
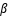
1 and
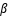
2 subbands generally across the cortex (Supplementary Figure 4). The signal structure in other (
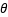
, α) EEG bands was characterized by non-stationarity of developing processes (a > 1). At the same time, the oscillations in the T3 (left anterior temporal) and O2 (right occipital) areas demonstrated Brownian noise (α~3/2) structure in the α1 subband.

Unlike Group S, the EEG data obtained from the participants in Group M during exposure to ammonia were characterized by the formation of an increased level of PSD value in the α3 subband (>0.2 N.U.) with a maximum in the central parietal (Pz) and right-hemisphere TPO regions. In addition, a particular increase in PSD value (>0.15 N.U.) was also observed in the O1-Pz area in the α2 subband, and bilaterally in the temporal and frontocentral cortical regions in the
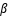
1 subband of EEG oscillations. The minimum PSD values were observed in the EEG spectrum's
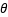
2 and
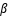
2 frequency bands. (Supplementary Figure 6)


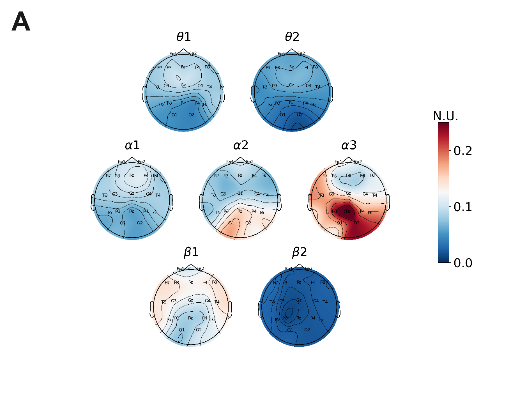


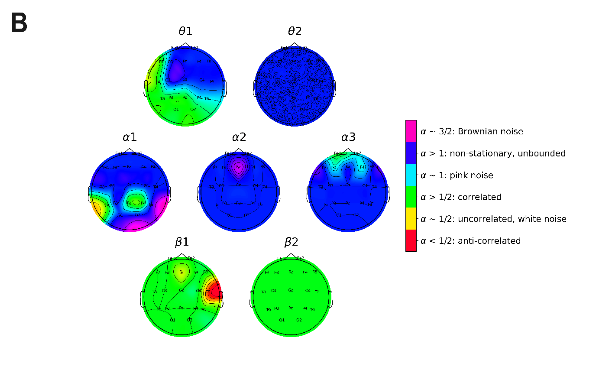


**Supplementary Figure 6. Topographical distribution of (A) PSD values, (B) α scaling exponent of DMA, calculated for the EEG data of the patients with moderate course of COVID-19 disease (Group M, n=21) during exposure to ammonia smell sample (trigeminal stimulus). Each scalp map corresponds to one EEG frequency subband: θ1, θ2, α1, α2, α3, β1, and β2. The color gradient represents (A) the values of the PSD coefficient and (B) the magnitude of the DMA scaling exponent.**

The use of the DMA demonstrated the EEG signal structure to be correlated and coherent in
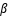
1,
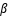
2, and
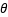
1 subbands (α > 1/2); the exceptions were detected for the T4 (right anterior temporal) area in the b1-subband, where the anticorrelated state of the signal (α < ½) was observed. Apart from that, the central-left hemispheric frontal-central-temporal region in the
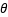
1 subband of the EEG oscillations, the neurodynamics was characterized by non-stationarity (α > 1). In addition, EEG processes in
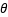
2 and α1-3 subbands were characterized by nonstationarity, except for the Fz lead (central frontal area) in the α2 subband (Brownian noise, α~3/2) and frontal and prefrontal regions in α3 subband (correlated processes, α > ½ ). Lastly, in the α1 subband, the occipital and posterior temporal areas of the right hemisphere (O2, T6) were characterized with Brownian noise-level of α-coefficient values, and central and right parietal regions (Pz-P4) demonstrated correlated EEG signal structure. A few oscillation types were observed in the left posterior temporal area (T5), from a stable coherent to an anticorrelated state (Supplementary Figure 6).

The perception of the trigeminal stimulus (ammonia) by the participants in Group H unexpectedly was characterized by the formation of a maximum PSD values focus in the α2 EEG subband in the posterior (left occipital and right TPO areas) and anterior – bilateral prefrontal and left posterior frontal (Fp1, Fp2, F7) cortical regions (0.2-0.3 N.U.). The PSD values in the remaining EEG bands were weakly expressed (<0.1 N.U.). However, a slight increase of the PSD value was observed in the following oscillatory subbands: in the anterior central area for the
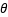
1 subband, in the right anterior frontal (F4) for the
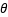
2 subband, and in the temporo-parietal-occipital area for the α3 subband (>0.1 N.U.). It should be noted that the lowest level of PSD was observed in the
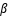
2-subband, generally across the cortex (<< 0.01 N.U.) (Supplementary Figure 7).

**
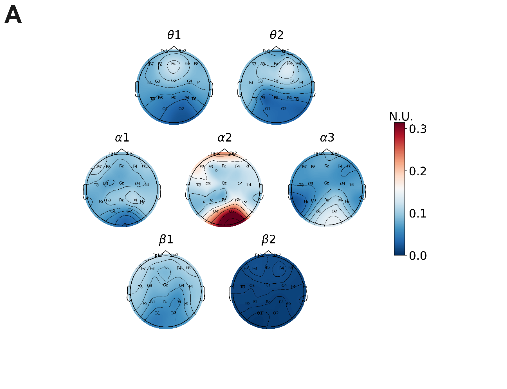
**


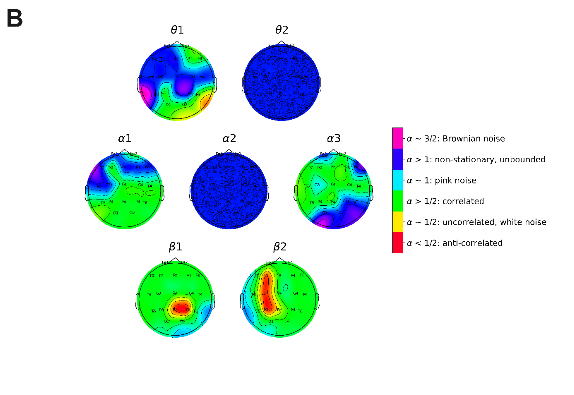


**Supplementary Figure 7. Topographical distribution of (A) PSD values, (B) α scaling exponent of DMA, calculated for the EEG data of the healthy control group (Group H, n=10) during exposition to ammonia smell sample (trigeminal stimulus). Each scalp map corresponds to one EEG frequency subband: θ1, θ2, α1, α2, α3, β1, and β2. The color gradient represents (A) the values of the PSD coefficient and (B) the magnitude of the DMA scaling exponent.**

In contrast to the topographical distribution of the PSD value over the cortex, the DMA results’ distribution was more diverse in terms of the EEG structure types representation (Supplementary Figure 7). Thereby, oscillatory processes in
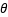
2 and α3 subranges were characterized by total non-stationarity (α > 1). In the
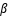
1,
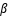
2, α1, and α3 subbands, the signal tends to correlate (coherent) (α>1/2) generally across the cortex. Exceptions were seen in the central and right anterior parietal areas (Pz-P4) for the b1-subbands and in fronto-centro-parietal regions of the left hemisphere (F3-C3-P3) for the b2-subband, where EEG processes had anticorrelation properties with elements of white noise (α<~1/2). In addition, in the right TPO within the
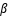
1 oscillatory subband and left temporal area in the
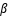
2 subband, the processes were characterized as non-stationary and partly tending to pink noise (α>=1/2). Within the α1 subband, the left temporal and bilateral prefrontal areas were characterized by non-stationarity (α >1), and the left posterior frontal region (F7) by Brownian noise (α=3/2). At the same time, the α3 subband oscillations demonstrated non-stationarity of neurodynamics (α>1) in the left prefrontal (Fp1), occipital (O1), TPO areas, and right posterior frontal region (F8); and Brownian noise levels of a coefficient (α=3/2) in the left occipital (O1) and bilateral posterior temporal (T5, T6) regions. Lastly, while the
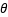
1 subband of the EEG data in the right frontal area, as well as in the C3, P3, O1, O2, Pz, P4, and T4 leads, the processes tended to correlate, anticorrelated oscillations characterized the posterior temporal region (T6)

Statistical analysis of the within-group changes in EEG activity during ammonia stimulation (Supplementary Figures 8,9) revealed distinct patterns across the three experimental cohorts (Supplementary Figures 8–9). In the severe COVID-19 group (Group S), exposure to the trigeminal stimulus elicited a significant increase in spectral power within the α2 subband, predominantly localized over midline and posterior cortical regions. This suggests an engagement of sensory-integrative networks under trigeminal activation. However, no other frequency bands demonstrated statistically significant power modulation relative to the resting state. Correspondingly, no significant changes in the DMA scaling exponent were observed in Group S, indicating stability in long-range temporal correlations under stimulation.

In contrast, participants in the moderate recovery group (Group M) showed no statistically significant alterations in either PSD or DMA indices during ammonia exposure compared to the resting baseline. Similarly, healthy controls (Group H) did not exhibit significant intra-condition changes in EEG spectral power or DMA parameters in response to the stimulus. Collectively, these findings indicate a lack of robust dynamic reorganization within Groups M and H during trigeminal stimulation, with only Group S showing localized reactivity in α2 oscillatory power.

**
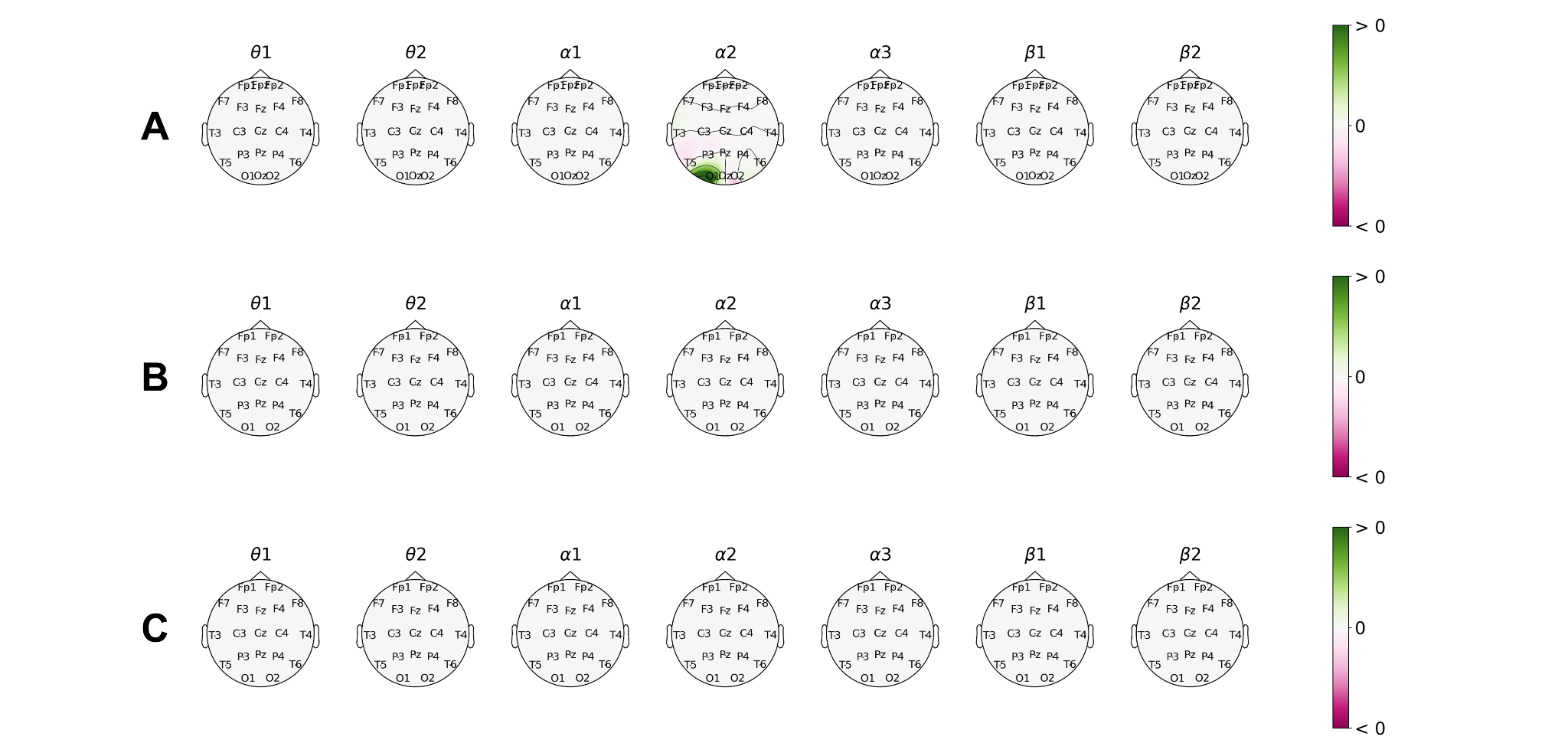
**

**Supplementary Figure 8. The topographical distributions of statistical differences in EEG power spectral density (PSD) between the resting state and the olfactory stimulation condition (ammonia administration), separately for each group: (A) participants with severe COVID-19 (Group S, n=20), (B) participants with moderate COVID-19 in recovery (Group M, n=21), and (C) healthy controls (Group H, n=10). The data were analyzed using the Mann–Whitney U test for each EEG frequency subband: θ1, θ2, α1, α2, α3, β1, and β2. The color scale represents the direction and magnitude of significant PSD changes: positive values (green hues) indicate an increase in power during olfactory stimulation relative to the resting state, while negative values (purple hues) reflect a power decrease, *p* < 0.05.
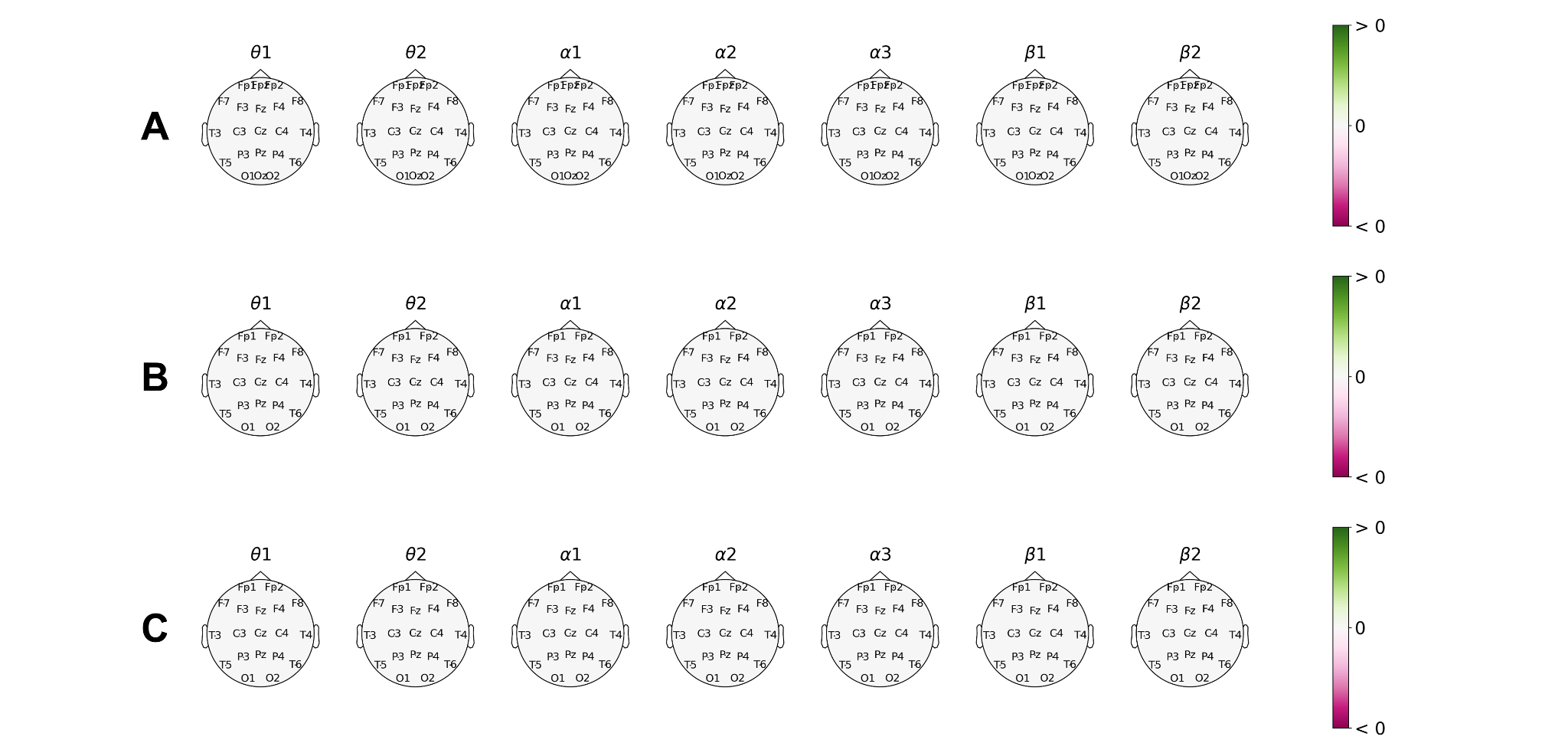
**

**Supplementary Figure 9. The topographical maps of statistically significant differences in EEG signal structure, assessed using detrended moving average (DMA) analysis, comparing the resting state and the olfactory stimulation condition (ammonia administration). Data are presented separately for (A) participants with severe COVID-19 (Group S), (B) participants with moderate COVID-19 in recovery (Group M), and (C) healthy controls (Group H). Each scalp map corresponds to one EEG frequency subband: θ1, θ2, α1, α2, α3, β1, and β2. The statistical comparisons were performed using the Mann–Whitney U test. The color gradient represents the direction and magnitude of the differences in DMA scaling exponents: Green hues (>0) indicate an increase in signal autocorrelation structure (i.e., stronger long-range temporal correlations) during olfactory stimulation, whereas Magenta hues (<0) reflect a decrease in such structuring, *p* < 0.05.**

Intergroup comparisons during ammonia exposure (Supplementary Figure 10) revealed marked differences in both neural activity's power spectral and temporal structuring. In terms of PSD, the severe COVID-19 group (Group S) demonstrated significantly elevated β2 power over bilateral temporo-parietal regions relative to both Group M and Group H, suggesting an upregulation of cortical excitation or compensatory hyperactivity in response to trigeminal input.

DMA-based analysis further revealed widespread increases in scaling exponent values in Group S, particularly within low-frequency subbands. Specifically, θ2 DMA values were significantly elevated in anterior cortical regions—including frontal, central, and anterior temporal areas—while θ1 subband increases were observed in left parietal and right occipital regions. A generalized elevation in α2 DMA values was also noted across the scalp, excluding the central-right parietal and right posterior frontal zones. These findings are indicative of globally heightened temporal autocorrelation and reduced signal complexity in Group S, consistent with dysregulated cortical processing.

No frequency subbands showed dominance by healthy controls (Group H), as reflected by the absence of green-coded areas in the scalp maps. Isolated blue-coded regions in β1 and α2 subbands suggest moderate illness participants (Group M) exhibited localized enhancements. However, these did not approach the spatial or statistical extent observed in Group S.

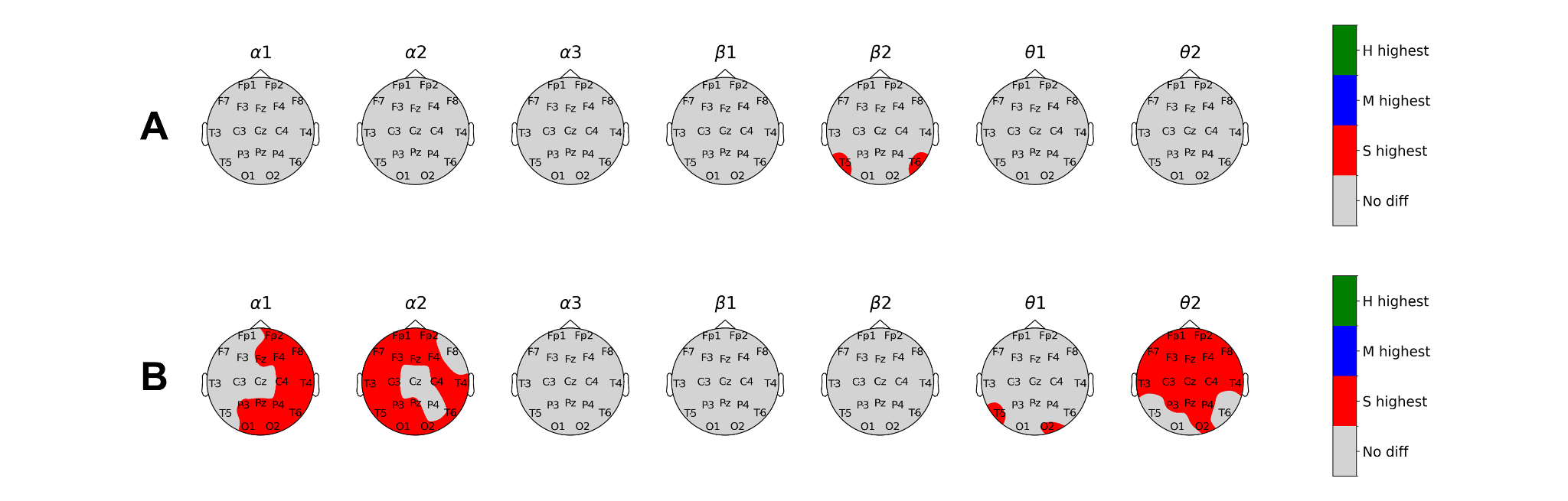


**Supplementary Figure 10. The topographical distribution of statistically significant differences in EEG power spectral density (PSD; Panel A) and detrended moving average (DMA; Panel B) across the three groups: individuals with severe COVID-19 (S), moderate COVID-19 (M), and healthy controls (H)—during olfactory stimulation with ammonia. The data were analyzed using the non-parametric Kruskal–Wallis H test. Each subplot represents one EEG subband: α1, α2, α3, β1, β2, θ1, and θ2. The colored regions on each scalp topography indicate the group showing the highest value for that frequency band and electrode. The legend (right) defines the color scheme: green (healthy controls (H) highest), blue (moderate COVID-19 group (M) highest), red: severe COVID-19 group (S) highest; Gray (no significant differences), *p* < 0.05.**

**3.** **Isoamyl Acetate**

The following agent tested in our studies was the odor of isoamyl acetate, known to be a primarily olfactory irritant. In our experiment, the olfactory stimulation in Group S patients caused a significant increase of the PSD (>> 0.2 N.U.) in the
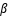
1 band oscillations bilaterally in the temporal areas of the cortex (Supplementary Figure 11): in the posterior regions of the cortex of the left hemisphere, and centrotemporal areas in the right hemisphere, respectively (Supplementary Figure 10). In addition, a slight increase in PSD was also noted in the
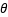
1 subband located in the right frontal-central-anterotemporal region (<0.15 N.U.) (Supplementary Figure 11). The minimum PSD values were found in the
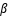
2 and
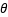
2 EEG subbands in almost all cortical areas, except for the posterior temporal regions in the
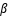
2 subband and frontocentral regions in the
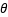
1 subband (~0.15 N.U).

**
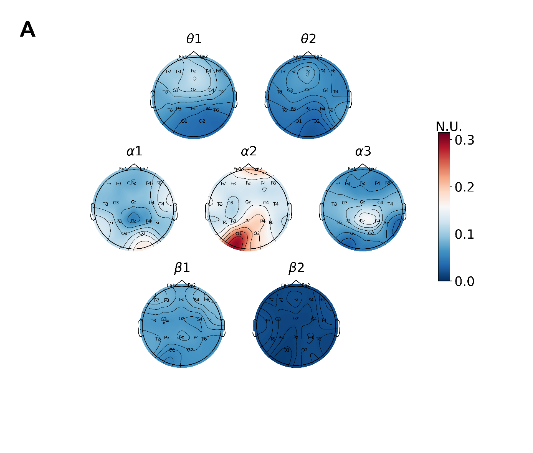
**


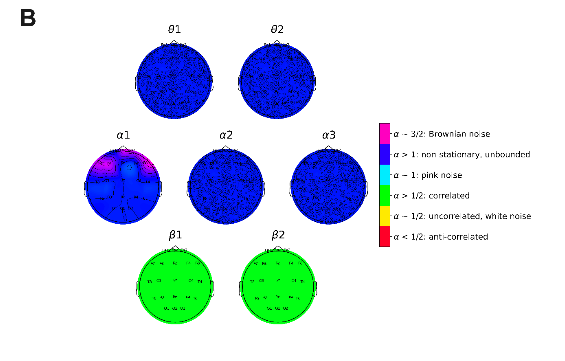


**Supplementary Figure 11/ Topographical distribution of (A) PSD values, (B) α scaling exponent of DMA, calculated for the EEG data of the patients with severe course of COVID-19 disease (Group S, n=20) during exposition to isoamyl acetate smell sample (olfactory stimulus). Each scalp map corresponds to one EEG frequency subband: θ1, θ2, α1, α2, α3, β1, and β2. The color gradient represents (A) the values of the PSD coefficient and (B) the magnitude of the DMA scaling exponent.**

An interesting and controversial fact, namely the low variability of characteristics, was obtained due to the DMA application (Supplementary Figure 11). The analysis of the EEG data in group S revealed that the
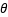
1,
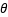
2, α1, α2, and α3 subbands were characterized by non-stationary processes (α>1) except for the frontal areas, where the EEG structure reached Brownian noise levels (α ~ 3/2).

The EEG parameters of patients who recovered from COVID-19 (Group M) had a slightly different spatial distribution. In this case, a significant enhancement in the PSD values was observed in the α3 subband within the right occipital and posterior temporal areas, as well as in the left temporal and posterior central regions of the cortex (>>0.2 N.U.) (Supplementary Figure 12). Moreover, an additional focus of significant PSD value elevation was observed in the occipital region within the α2 subband of the EEG (~ 1.5 N.U.). At the same time, the level of activation in the
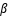
1 and
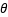
1 subbands was characterized by average PSD values (~0.1 N.U.), with a slight increase in the T5, F4-F8, and T6 leads in the
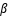
1 subband (~1.5 N.U.). The minimal PSD values were observed in the
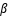
2 and
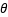
2 EEG subbands in almost all cortical leads. (<< 0.1 N.U.) (Supplementary Figure 12).

**
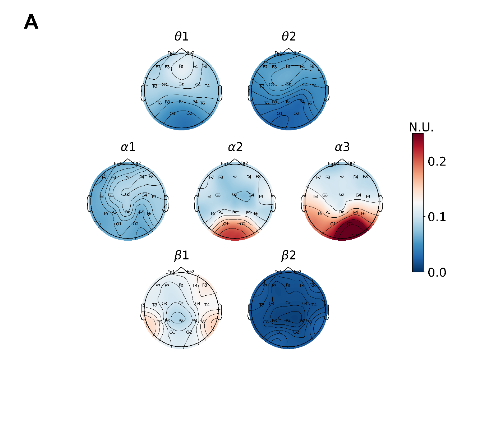
**

**
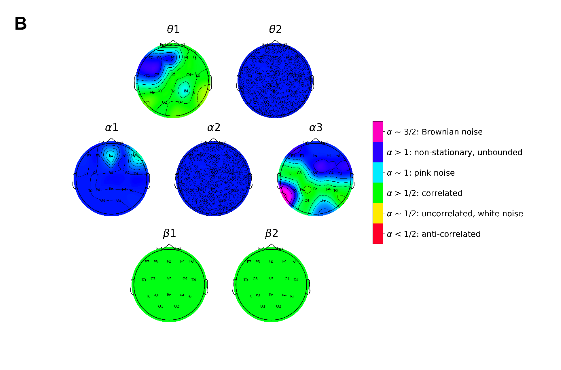
**

**Supplementary Figure 12. Topographical distribution of (A) PSD values, (B) α scaling exponent of DMA, calculated for the EEG data of the patients with moderate course of COVID-19 disease (Group M, n=21) during exposure to isoamyl acetate smell sample (olfactory stimulus). Each scalp map corresponds to one EEG frequency subband: θ1, θ2, α1, α2, α3, β1, and β2. The color gradient represents (A) the values of the PSD coefficient and (B) the magnitude of the DMA scaling exponent.**

The DMA analysis for the EEG data obtained from Group M revealed that the oscillatory processes in the
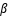
1 and
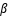
2 were highly coherent (α> 1/2). Similar neurodynamical patterns were observed in the
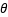
1 subband, except for frontal, anterior temporal, and central (Fz, F3, F7, T3, C3) areas of the left hemisphere, where the signal was in a non-stationary state (α>1), and right anterior parietal region (Р4), where a coefficient was at the level of pink noise (α=1). The pink noise properties of electrogenesis were also detected in the central frontal (Fz) and right posterior frontal (F8) areas within the α1 subband. Lastly, in the α3 subband, the non-stationary processes in the anterior-central areas bilaterally were accompanied by the development of correlation noise (α> 1/2) signal properties in the right occipital (O2)and the left posterior temporal (T5) areas (Supplementary Figure 12).

In the control Group H subjects, olfactory activation by isoamyl acetate was characterized by a pronounced focus of maximum activity in the α2 subband, localized in the left occipital zone (~ 0.3 N.U.). In addition, within this electrical subband, synchronization of oscillations of moderate power (<0.2 N.U.) was observed in the central parietal and lower frontal areas of the cortex bilaterally. Minimum values (0.1-0.2 N.U.) of PSD were observed in
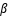
2,
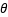
2,
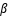
1, α3,
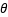
1, and EEG subbands. The exceptions were noted in the central-parietal cortical areas within the α3 subband, the central anterior zone within the
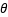
1 subband, the right temporal and occipital, and the left posterior-temporal areas within the α1 subband, where the power coefficients had relatively average values (~0.1-0.2 N.U.) (Supplementary Figure 13).


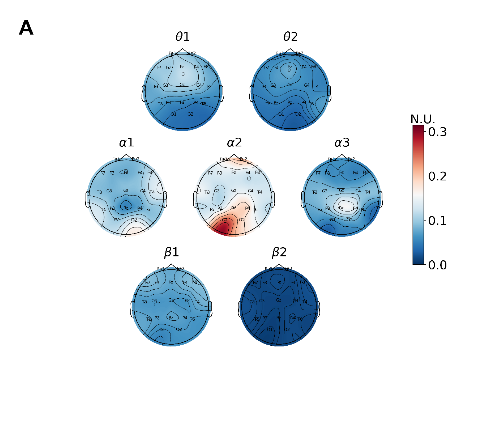


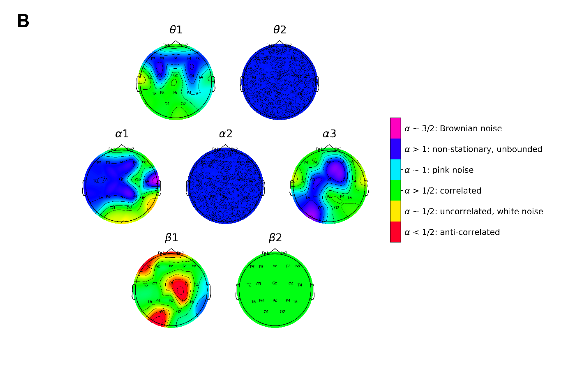


**Supplementary Figure 13. Topographical distribution of (A) PSD values, (B) α scaling exponent of DMA, calculated for the EEG data of the healthy control group (Group H, n=10) during exposition to isoamyl acetate smell sample (olfactory stimulus). Each scalp map corresponds to one EEG frequency subband: θ1, θ2, α1, α2, α3, β1, and β2. The color gradient represents (A) the values of the PSD coefficient and (B) the magnitude of the DMA scaling exponent.**

As for the DMA, it turned out that the oscillatory EEG dynamics in Group H were overall non-stationary (α> 1) in the
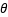
2,
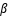
2 subbands; such signal properties were also observed bilaterally in the prefrontal and central areas of the cortex within the
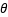
1 subband, along with left temporal-prefrontal area and several other leads, such as central (Cz), right anterior frontal, parietal and temporal regions (F4, P4, T4). The non-stationarity of oscillation processes was also observed in some cortex regions in the α3 and
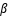
1 subbands. In the α3 subband, this area included left occipital, posterior temporal, anterior parietal, and central leads (O1, T5, P3, C3), frontal central (Fz), and right anterior (F4) leads. Within the
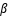
1 subband, non-stationary oscillations were observed in the right posterior temporal area (T6). It should be noted that oscillatory processes in the areas of their non-stationarity merged into areas with activity, which was characterized as "pink noise" (α=1). In addition, the Brownian noise locus (α=3/2) was also observed in the right anterior temporal area (T4) (Supplementary Figure 13).

At the same time, highly correlated neurodynamics (α >1/2) (Supplementary Figure 13) were observed in the following cortical areas and EEG-subbands: in the
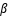
2 subband generally across the cortex; in
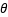
1 subband within the occipital, frontal, and parietal areas bilaterally, central parietal and right temporal regions; in α1 subband within the bilateral anterior frontal (F3, F4), right posterior frontal (F4), central (C4), bilateral anterior parietal (P3, P4) and left posterior temporal (T5) areas; in α3 subband within the left hemispheric frontal and temporal regions and right hemispheric temporal, parietal and occipital zones of the cortex; in
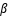
1 subband within the bilateral frontal areas (F3, Fz, F4, F8), bilateral central (C3, C4), anterior parietal (P3, P4), occipital (O1, O2), and left temporal (T3) areas. It is also noteworthy that within the
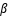
1 subband in the left occipital-parietal and posterior frontal areas, in addition to the bilateral inferior frontal regions, as well as central (Cz) and right central-parietal (C4, P4) regions, the development of anti-correlated, incoherent oscillations was observed (white noise) (α<½).

Intragroup comparisons between resting state and isoamyl acetate stimulation (Supplementary Figures 14–15) revealed no statistically significant changes in EEG power spectral density (PSD) or detrended moving average (DMA) scaling exponents within any of the study groups.

In the severe COVID-19 group (Group S), although visual inspection of scalp topographies indicated elevated α2 and α3 activity during stimulation, primarily over posterior and temporal regions, these changes did not reach statistical significance when formally tested against baseline. Similarly, the moderate recovery group (Group M) did not exhibit significant PSD or DMA alterations in response to the olfactory stimulus, despite localized increases in α3 and β1 power visible in individual topographies. The healthy control group (Group H) also showed no significant modulation in oscillatory power or signal autocorrelation structure across any frequency band during isoamyl acetate exposure.

These results suggest that isoamyl acetate, as a predominantly olfactory stimulus, elicited no robust reorganization of cortical dynamics within the examined groups under passive stimulation conditions, contrasting with the more reactive α2 response observed during trigeminal input in Group S.


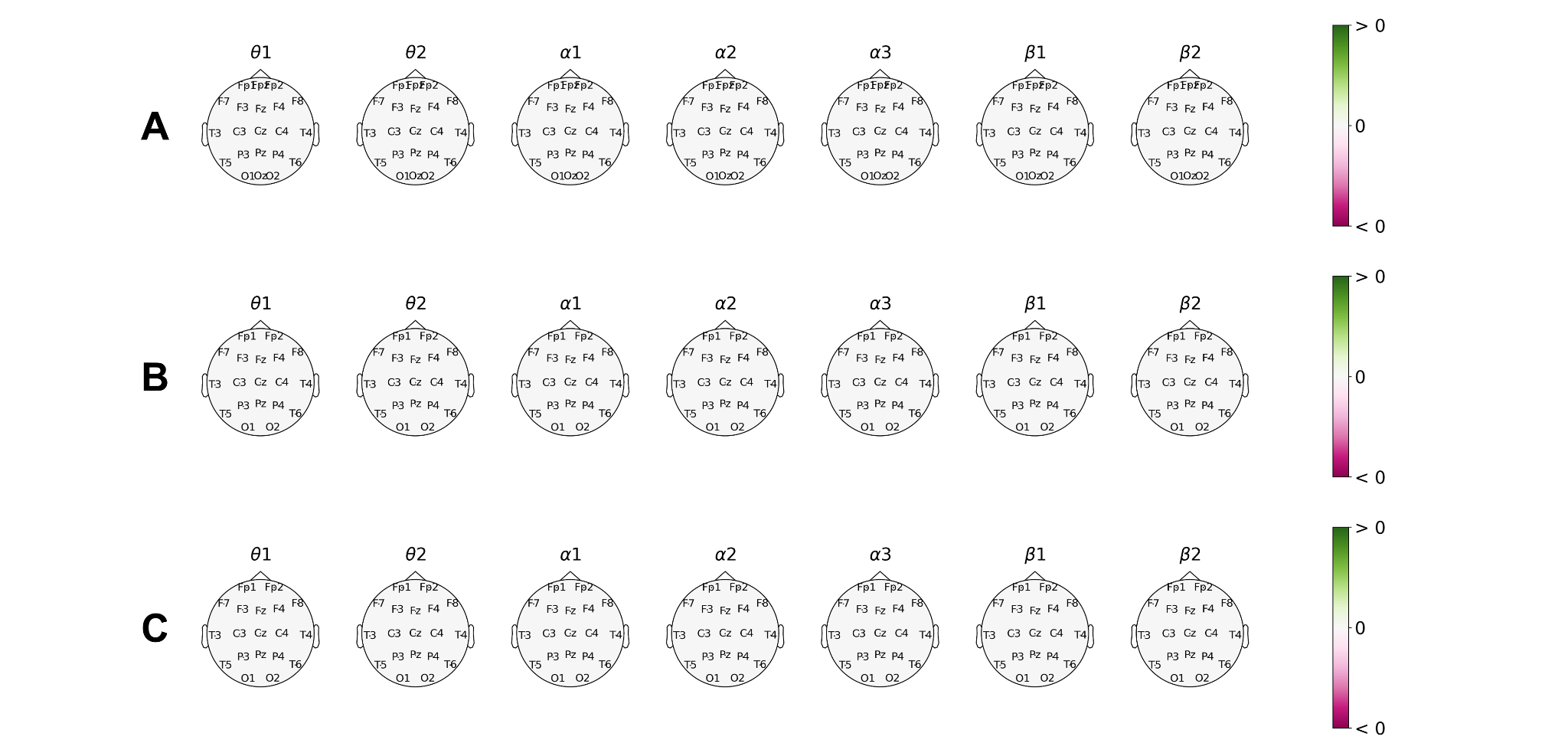


**Supplementary Figure 14. The topographical distributions of statistical differences in EEG power spectral density (PSD) between the resting state and the olfactory stimulation condition (isoamyl acetate administration), separately for each group: (A) participants with severe COVID-19 (Group S, n=20), (B) participants with moderate COVID-19 in recovery (Group M, n=21), and (C) healthy controls (Group H, n=10). The data were analyzed using the Mann–Whitney U test for each EEG frequency subband: θ1, θ2, α1, α2, α3, β1, and β2. The color scale represents the direction and magnitude of significant PSD changes: positive values (green hues) indicate an increase in power during olfactory stimulation relative to the resting state, while negative values (purple hues) reflect a power decrease, *p* < 0.05.**

**Supplementary Figure 15. The topographical maps of statistically significant differences in EEG signal structure, assessed using detrended moving average (DMA) analysis, comparing the resting state and the olfactory stimulation condition (isoamyl acetate administration). Data are presented separately for (A) participants with severe COVID-19 (Group S), (B) participants with moderate COVID-19 in recovery (Group M), and (C) healthy controls (Group H). Each scalp map corresponds to one EEG frequency subband: θ1, θ2, α1, α2, α3, β1, and β2. The statistical comparisons were performed using the Mann–Whitney U test. The color gradient represents the direction and magnitude of the differences in DMA scaling exponents: Green hues (>0) indicate an increase in signal autocorrelation structure (i.e., stronger long-range temporal correlations) during olfactory stimulation, whereas Magenta hues (<0) reflect a decrease in such structuring, *p* < 0.05.**

The statistical analysis of data obtained from the three experimental groups (Supplementary Figure 16) highlighted that the severe group (S) showed elevated oscillatory power in the β1 and β2 bands across central and temporo-parietal regions bilaterally, suggesting heightened cortical excitation during stimulation. On the other hand, based on the DMA findings, the S group consistently exhibited significantly higher DMA values across almost all frequency bands and electrodes, particularly in the α1, α2, θ1, and θ2 subbands, pointing to increased temporal autocorrelation or rigidity in neural dynamics. Within the α1 subband, elevated values were distributed generally over the cortex, except the left anterior frontal and right posterior frontal areas. Within the α2 subband, elevated values were distributed predominantly over the anterior regions of the left hemisphere (fronto-central-temporal); within the frontal areas, DMA elevation was bilateral, and high scaling exponent values were observed in the left occipital cortical area. The θ1 subband, on the other hand, demonstrated high DMA values in the right posterior frontal and temporal areas, and within frontal and occipital areas bilaterally. Lastly, within the θ2 scaling exponent, values were the highest for the S Group all over the cortex, except for the left central and bilateral parietal cortex areas.

**Supplementary Figure 16. The topographical distribution of statistically significant differences in EEG power spectral density (PSD; Panel A) and detrended moving average (DMA; Panel B) across the three groups: individuals with severe COVID-19 (S), moderate COVID-19 (M), and healthy controls (H)—during olfactory stimulation with isoamyl acetate. The data were analyzed using the non-parametric Kruskal–Wallis H test. Each subplot represents one EEG subband: α1, α2, α3, β1, β2, θ1, and θ2. The colored regions on each scalp topography indicate the group showing the highest value for that frequency band and electrode. The legend (right) defines the color scheme: green (healthy controls (H) highest), blue (moderate COVID-19 group (M) highest), red: severe COVID-19 group (S) highest; Gray (no significant differences), *p* < 0.05.**

**4.** **Mountain Pine**

We also studied the perception of a complex ester (olfactory-trigeminal) agent - the smell of mountain pine essential oil. Examination of patients from Group S revealed that perception of the pine smell was accompanied by a significant PSD increase in the 1 subband with a focus of maximum activity in the left temporal and parietal areas (T3, T5, P3) (>> 0.20 N.U.) (Supplementary Figure 17). In addition, in the 2 subband, an increased level of PSD (0.15-0.2 N.U.) was also observed in the right temporal-parietal-central areas and the left anterior temporal area, but without a pronounced maximum. The minimum PSD values were observed in the 2, 2, and α3 subbands. In the central anterior cortical area, a focus of elevated PSD value was also observed against the background of general levels distributed over the scalp (~0.05 N.U) in the 1 and α1 subbands (Supplementary Figure 17).

**Supplementary Figure 17. Topographical distribution of (A) PSD values, (B) α scaling exponent of DMA, calculated for the EEG data of the patients with severe course of COVID-19 disease (Group S) during exposure to mountain pine (*p. mugo*) essential oil smell sample (mixed stimulus)**

At the same time, the DMA revealed the following: the processes of cortical electrogenesis within the high-frequency part of the EEG data spectrum (1, 2) were in a highly correlated coherent state (α> 1/2), while EEG oscillations in other ranges were in a non-stationary, unstable, chaotic state (α>1) (Supplementary Figure 16). To a certain extent, the exception was marked in the α1 subband of the EEG. Here, in the left frontal-temporal-parietal areas (F3, F7, T3, P3, Pz), the EEG dynamics were described as Brownian noise (α~3/2). In addition, in the same subband within the left temporal and bilateral occipital areas of the EEG, the processes fell in the range of pink noise (hierarchical complexity of information processes).

In contrast to patients in Group S and Group M, a center of significant α3-synchronization was formed bilaterally in the occipital, parietal, and posterior temporal regions (T5, P3, O1, Pz, O2, P4, T6) (>0.2 N.U) (Supplementary Figure 18). In addition, an increased level of PSD was noted in the occipital area within the α2 subband and the left frontal-temporal area within the α1 subband (>0.15N.U.). The minimum values of the relative power of EEG oscillations were observed in 2-, 2, and 1 (except for the central leads), 1, and α1 subbands (~0.1 N.U.) (Supplementary Figure 18).

**Supplementary Figure 18. Topographical distribution of (A) PSD values, (B) α scaling exponent of DMA, calculated for the EEG data of the patients with moderate course of COVID-19 disease (Group M) during exposure to mountain pine (*p. mugo*) essential oil smell sample (mixed stimulus)**

At the same time, the use of DMA revealed the presence of a total (generalized over scalp) correlation (coherence) of EEG dynamics in the so-called cognitive oscillatory subbands (1,1, 2) and frontal temporal regions in the α3 subbands (F7, F3, F4, Fz, F8, T4, C4) (α>1/2). In addition, it was found that the EEG processes in the left frontal-parietal (F3, Pz) area within the 2 subband and right temporal (T6) region within the α3 subband were characterized as Brownian noise (accumulation of information) (α=3/2). The right posterior frontal (F7) area was marked as an area of pink noise propagation (complex information) (α~1) (Supplementary Figure 18).

The topographical distribution of PSD values in the EEG data of the patients from Group H was characterized by the presence of a focus of increased PSD values in the α2 subband and the synchronization of oscillations in the α1 subband located in the left hemispheric part of the posterior temporal-parietal areas. The remaining subbands were characterized by the minimum values of the PSD (Supplementary Figure 18).

**Supplementary Figure 19. Topographical distribution of (A) PSD values, (B) α scaling exponent of DMA, calculated for the EEG data of the healthy control group (Group H) during exposition to mountain pine (*p. mugo*) essential oil smell sample (mixed stimulus)**

The DMA method showed a rather complex architecture of the EEG data oscillatory processes. Non-stationarity (α>1) of processes was noted in the 2 and α2 subbands almost generally all over the scalp, as well as in the 1 subband within the left frontal, right temporal, central parietal, central, and left occipital zones. Moreover, in the α1 subband, such signal properties were observed in the left frontal-temporal-occipital area. The central-frontal-right anterior-temporal zone, in α3 subband – within the central-frontal and anterior frontal-parietal zones bilaterally (Supplementary Figure 19). Correlated (α > 1/2) (coherent) EEG dynamics were observed in the left temporal, central, right frontal and right posterior cortical areas in the 1 subrange, generally across the cortex in the α3 subband (except for the bilateral temporal and central areas); in the 1 subband, such dynamics were observed across the cortex (except for the occipital and posterior temporal regions bilaterally), in the 2 subband- within the right posterior and left anterior frontal regions (Supplementary Figure 18).

It should be noted that high-frequency EEF subbands ( 1,2) were characterized by the presence of areas with anti-correlated signal properties (α<1/2) – posterior regions of the left hemisphere (O1, T5, P3, Pz) in 1 subband, and the left-frontal-temporal-central, and the right temporal zones in 2 subband (Supplementary Figure 18). It is also necessary to note the Brownian noise-level activity foci, located in the left occipital and posterior temporal areas within the α1 subband, the right temporal area in α2 subband and left frontal-temporal region (including Fz) in α3 subband.

Statistical evaluation of intragroup EEG dynamics during exposure to mountain pine essential oil revealed no significant changes in either power spectral density (PSD) or detrended moving average (DMA) scaling exponents when compared to the resting state baseline (Supplementary Figures 20–21).

In the severe COVID-19 group (Group S), however, topographical patterns displayed apparent increases in PSD within the α2, α3, and β1 subbands—particularly over central and parieto-occipital regions—but these enhancements did not reach statistical significance at the group level. Similarly, visual inspection suggested modulation of low-frequency bands in the DMA exponent maps, especially in the θ1 subband; however, these trends were not supported by formal testing.

The moderate COVID-19 recovery group (Group M) and healthy controls (Group H) exhibited stable electrophysiological profiles under pine stimulation. Despite individual variations and localized oscillatory changes, such as α3 synchronization in posterior cortical regions of Group M, there were no statistically significant differences between rest and stimulation conditions in either PSD or DMA metrics. These findings indicate an absence of robust within-group neurodynamic shifts under mixed olfactory-trigeminal load.

**Supplementary Figure 20. The topographical distributions of statistical differences in EEG power spectral density (PSD) between the resting state and the olfactory stimulation condition (pine scent administration), separately for each group: (A) participants with severe COVID-19 (Group S, n=20), (B) participants with moderate COVID-19 in recovery (Group M, n=21), and (C) healthy controls (Group H, n=10). The data were analyzed using the Mann–Whitney U test for each EEG frequency subband: θ1, θ2, α1, α2, α3, β1, and β2. The color scale represents the direction and magnitude of significant PSD changes: positive values (green hues) indicate an increase in power during olfactory stimulation relative to the resting state, while negative values (purple hues) reflect a power decrease, *p* < 0.05.**

**Supplementary Figure 21. The topographical maps of statistically significant differences in EEG signal structure, assessed using detrended moving average (DMA) analysis, comparing the resting state and the olfactory stimulation condition (pine scent administration). Data are presented separately for (A) participants with severe COVID-19 (Group S), (B) participants with moderate COVID-19 in recovery (Group M), and (C) healthy controls (Group H). Each scalp map corresponds to one EEG frequency subband: θ1, θ2, α1, α2, α3, β1, and β2. The statistical comparisons were performed using the Mann–Whitney U test. The color gradient represents the direction and magnitude of the differences in DMA scaling exponents: Green hues (>0) indicate an increase in signal autocorrelation structure (i.e., stronger long-range temporal correlations) during olfactory stimulation, whereas Magenta hues (<0) reflect a decrease in such structuring, *p* < 0.05.**

Intergroup comparisons during mountain pine stimulation revealed pronounced between-group disparities in both spectral amplitude and temporal organization of neural activity (Supplementary Figure 22). The severe COVID-19 group (Group S) exhibited dominant PSD values in the β2 subband, with broad spatial distribution covering central and posterior cortices. This suggests a pattern of sustained cortical excitation, potentially reflecting hyperresponsivity to multimodal sensory input.

In the DMA domain, Group S again demonstrated significantly elevated α-scaling exponents across the majority of frequency bands and cortical regions. Specifically, within the α1 subband, increased scaling exponents were localized to anterior frontal and right parieto-temporal areas, suggesting altered frontal-limbic coupling. For the α2 subband, elevated values were observed across nearly the entire cortex, excluding anterior frontal sites, indicating widespread autocorrelation and possible rigidity in higher-order information integration. The θ1 demonstrated enhanced temporal structuring in anterior frontal, central, and left temporal areas. For the θ2 subband, peak DMA values were concentrated in left fronto-temporo-parietal, midline central, and right centro-temporal areas, reinforcing the observation of global signal structuring and decreased adaptability.

The moderate illness group (Group M) showed limited focal increases in PSD, most notably in the α3 band over posterior areas, and exhibited some isolated enhancements in DMA values in β1 and θ1 subbands. In contrast, the healthy control group (Group H) did not show topographic dominance in any frequency band, as evidenced by the near-total absence of green-coded regions in the intergroup scalp maps.

Together, these results highlight the persistent cortical hypersynchrony and elevated temporal rigidity in Group S during multisensory stimulation, in contrast to the more regionally restricted and physiologically typical patterns observed in Groups M and H. These intergroup differences further reinforce the hypothesis of ongoing cortical dysregulation in individuals with a history of severe COVID-19, even under complex sensory processing conditions.

**Supplementary Figure 22. The topographical distribution of statistically significant differences in EEG power spectral density (PSD; Panel A) and detrended moving average (DMA; Panel B) across the three groups: individuals with severe COVID-19 (S), moderate COVID-19 (M), and healthy controls (H)—during olfactory stimulation with pine scent. The data were analyzed using the non-parametric Kruskal–Wallis H test. Each subplot represents one EEG subband: α1, α2, α3, β1, β2, θ1, and θ2. The colored regions on each scalp topography indicate the group showing the highest value for that frequency band and electrode. The legend (right) defines the color scheme: green (healthy controls (H) highest), blue (moderate COVID-19 group (M) highest), red: severe COVID-19 group (S) highest; Gray (no significant differences), *p* < 0.05.**
